# BMP8A, TGF-β1 regulates chicken chondrocyte proliferation, differentiation, and apoptosis induced by Thiram

**DOI:** 10.5713/ab.25.0413

**Published:** 2025-09-30

**Authors:** Yuxiang Lu, Hengyong Xu, Xuyang Ji, Yuxin Zhou, Zhi Hu, Felix Kwame Amevor, Ranran Du, Xiaoling Zhao, Yiping Liu, Yan Wang

**Affiliations:** 1 Key Laboratory of Livestock and Poultry Multi-omics, Ministry of Agriculture and Rural Affairs, College of Animal and Technology (Institute of Animal Genetics and Breeding), Chengdu, China; 2 Farm Animal Genetic Resources Exploration and Innovation Key Laboratory of Sichuan Province, Sichuan Agricultural University, Chengdu, China; 3 State Key Laboratory of Swine and Poultry Breeding Industry, Sichuan Agricultural University, Chengdu, China

**Keywords:** *BMP8A*, Chicken Chondrocyte, Thiram, Tibial Dyschondroplasia, Transforming Growth Factor-β1

## Abstract

**Objective:**

Tibial dyschondroplasia (TD) is a metabolic disorder of cartilage that impairs the development of the tibial growth plate in rapidly growing poultry. This study aimed to identify key genes and clarify the molecular mechanisms involved in TD in broiler chickens. The study evaluated the potential effect of vitamin D_3_ (VD_3_) in alleviating TD symptoms, focusing particularly on the role of Bone morphogenetic protein 8A (BMP8A) and its interaction with transforming growth factor-β1 (TGF-β1).

**Methods:**

Ninety-four broiler chicks were allocated into three groups: healthy control, thiram-induced TD, and thiram-induced with VD_3_ supplementation. RNA sequencing was performed to identify differentially expressed genes (DEGs) among the groups. Target genes underwent additional validated using molecular biology techniques, such as gene expression analysis and *in vitro* functional assays on chondrocytes.

**Results:**

VD_3_ effectively mitigated chondrocyte damage induced by thiram. RNA-seq revealed 625 DEGs enriched in pathways such as the TGF-β signaling pathway. Four co-DEGs (*BMP8A*, *COL10A1*, *SDC3*, and *SCIN*) were closely associated with collagen metabolism and reorganization. Functional assays, such as CCK8, EdU and IHC showed that BMP8A reduced collagen accumulation induced by elevated TGF-β1 levels, promoted the release of collagen types I, II, and X, and facilitated chondrocyte proliferation and differentiation while reducing apoptosis.

**Conclusion:**

BMP8A plays a protective role in TD by the regulation of collagen balance and the maintenance of chondrocyte function, especially in the presence of high TGF-β1 levels. VD_3_ supplementation effectively reduces TD-related damage. The interaction between BMP8A and TGF-β1 may provide a novel therapeutic target for the prevention and treatment of TD in poultry.

## INTRODUCTION

The global demand for poultry protein has driven intensive breeding practices to improve broiler growth rates. However, accelerated growth often impairs skeletal integrity, leading to manifestations such as cartilage degeneration, reduced tibial shear strength, and tibial curvature, such as tibial dyschondroplasia (TD), an osteonosus characterized by masses of avascular cartilage in the tibial growth plate [1]. TD is a serious skeletal disease that causes pain, lameness, and limited mobility in chickens, resulting in chronic stress, thereby causing both significant economic losses and serious animal welfare problems in the poultry industry [2].

Thiram (tetramethyl thiuram disulfide), a widely used lipophilic fungicide in agriculture, has been implicated in the development of TD. Its administration induces pathological changes that mirror spontaneous TD, including avascular ossification zones and arrest of chondrocyte maturation arrest in tibial growth plates [3]. Notably, dietary supplementation with 100 mg/kg thiram reliably generates uniform TD models by recapitulating key histopathological features, such as cartilage thrombi and immature chondrocyte accumulation [3,4]. This approach addresses the challenge of inconsistent incidence of TD in natural settings, allowing for standardized experimental studies [5]. Despite its utility in research, early detection of TD remains problematic in commercial flocks due to subclinical manifestations, often leading to underestimation of its economic impact [6].

Recent studies have elucidated key mechanisms underlying TD, revealing complex interactions between genetic predispositions, molecular signaling, and environmental triggers. The TGF-β/BMP signaling axis has emerged as a critical regulator of osteochondral development, with extensive research establishing its dual role in bone homeostasis and pathology [7,8]. Skeletal expression patterns of TGF-β isoforms (β1, β2, β3) and specific BMP members (BMP2/4/6/7) demonstrate their essential functions in endochondral ossification and fracture repair [9,10]. Notably, BMP8A polymorphisms were first associated with ankylosing spondylitis susceptibility in Iranian populations, with subsequent validations [11,12]. Emerging evidence implicates additional regulatory networks in the pathogenesis of TD, such as miR-140-5p/HDAC4 interactions and OPG/RANKL axis modulation [13,14]. Vitamin D3 (VD_3_) shows particular promise through its influence on calcium-phosphate metabolism and hydroxyapatite formation [15]. Previous studies using INS-VD3/BMP2-FGF2 combinations show enhanced bone regeneration in diabetic models [16], while optimal VD_3_ levels correlate with improved skeletal integrity across species [17]. However, it remains uncertain to what extent this is applicable to TD in broiler chickens.

Lu et al [5] identified differential expressions of *BMP8A*, *Col Xα1*, *SCIN*, and *SDC3* in TD-affected chickens. Building on this foundation, the current study integrates the VD_3_ intervention in thiram-induced TD models with multi-omics analysis of growth plate cartilage. This systematic approach clarifies BMP8A/TGF-β1 regulatory mechanisms of BMP8A/TGF-β1 in TD progression while proposing new diagnostic biomarkers and therapeutic targets. The findings advance our understanding of avian skeletal disorders and inform strategies for genetic improvement in poultry breeding programs.

## MATERIALS AND METHODS

### Experimental design and animals

Ninety-four 1-day-old Qingjiaoma broilers (from the Sichuan Agricultural University Poultry Breeding Farm) were randomly divided into three groups on day 6: Control (n = 29, basal diet), Thiram (#C10036460; Macklin Biochemical) (n = 29, 100 mg/kg of thiram supplemented diet), and Thiram+ VD_3_ (n = 36, thiram diet+VD_3_ doses: 25 mg/kg, 50 mg/kg, 75 mg/kg, and 100 mg/kg) (Supplement 1). Diets were formulated according to breed-specific nutritional requirements (New Hope Group). On day 10, six birds per group were sacrificed by pentobarbital injection for bone index assessment. The tibial growth plates were fixed in 4% paraformaldehyde or frozen in liquid nitrogen (−80°C) for histological and transcriptomic analyzing. The chickens (n = 40) were then selected for subsequent chondrocyte culture from the control and the thiram group.

### Bone index quantification

The ties of the 10-day-old chicks were dissected and measured as follows: The weight and length (including or excluding the cartilage ends) of the tibia were measured using an analytical balance (Shimaszu, #AUW220D) and a vernier caliper (Mitutoyo). Bone index was calculated as cartilage weight/body weight×100%.

### Chondrocyte isolation and culture

Chondrocytes were extracted from the proximal tibia of 10-day-old Qingjiaoma broiler chickens, following the methods of Lu et al [5]. Initially, the periosteum and other organized structures were carefully removed, and approximately 1 mm^3^ fragments of cartilage were cut from the proximal tibia. These sections were treated with 0.25% trypsin (Gibco) (37°C, 20 min), followed by 0.1% collagenase II (Sigma-Aldrich) (37°C, 2.5 h). Chondrocytes cultured in Dulbecco’s modified Eagle Medium (DMEM)-F12 (Sigma-Aldrich) supplemented with 10% fetal bovine serum (FBS; Gibco) and 1% penicillin-streptomycin (Solarbio) at 37°C and 5% CO_2_. Chondrocytes from each group were harvested on days 4, 7, and 10 for quantitative real-time polymerase chain reaction (RT-qPCR) analysis.

### Quantitative real-time polymerase chain reaction

Total RNA was extracted using RNAiso plus Reagent (Takara), reverse-transcribed using PrimeScript RT reagent kit (Takara), and gene expression was detected on a LightCycler96 qPCR system (Roche). The RT-qPCR primer sequences for target genes (Supplement 2) are obtained from Lu et al [5] and Xu et al [18] or designed using NCBI. The reaction conditions were performed at 98°C for 4 min (predetermined), followed by 98°C for 15 s (denaturation), 60°C for 20 s (annealing), 72°C for 10 s (extension), duplicated 40 times. Relative mRNA levels were normalized to *GAPDH* using 2^−ΔΔCT^ method. All samples were performed in triplicate.

### Analysis of mRNA-seq, annotation, and enrichment

RNA-seg libraries were prepared from Thiram+VD3 (50 mg/kg) samples (2 ug total RNA/sample) and analyzed alongside previous datasets [5]. Functional annotation of differentially expressed genes (*BMP8A*, *Col10A1*, *SDC3*, *SCIN*) was performed via OmicShare tools ( https://www.omicshare.com/tools) for the enrichment of the GO, KEGG, and Reactome pathway.

### Chondrocyte transfection for BMP8A and pc-transforming growth factor-β1

Chondrocytes at 60%–70% confluence were transfected with siRNA (si-BMP8A variants, si-NC) (Supplement 3) or overexpression constructs (pc-NC, pc-BMP8A, pc-TGF-β1), designed and synthesized by Sangon Biotech, using the Lipofectamine 3000 kit (Invitrogen). Quantitative PCR was used to assess transfection efficiency. Each transfection was performed in triplicate.

### Chondrocyte vitality and proliferation assay

The viability of the chondrocytes was assessed using a CCK-8 assay (MeilunBio): chondrocytes were seeded in 96-well plates at a density of 3×10^3^ cells per well, followed by the addition of 10μL CCK-8 reagent to each well. The absorbance value (OD) at 450 nm was measured using a microplate reader (Thermo Fisher Scientific) at 12 h, 24 h, 36 h, 48 h. Each sample was analyzed in triplicate for accuracy and reliability. Additionally, cells were incubated in EdU medium at 37°C, 5% CO_2_ for 3 h, followed by staining with Apollo reagent in the dark for 20 min, then observed using fluorescence microscopy (DP80; Olympus). Triplicate biological replicates.

### Chondrocytes cycle and apoptosis analysis

Cells firstly fixed in 70% ethanol overnight at 4°C and then stained with propidium iodide (PI) for cycle analysis. Apoptosis was evaluated using Annexin V-FITC/PI dual staining (Vazume) following the manufacturer’s protocol. The results were analyzed using flow cytometry (BD Biosciences). All experiments were carried out in triplicate.

### Immunocytochemical and enzyme-linked immunosorbent assay analyzes of chondrocytes

Chondrocytes were transfected with si-BMP8A, si-NC, pc-BMP8A, or pc-NC for 48 h (60%–70% confluence) and processed for immunocytochemistry. Cells seeded on glass coverslips (Solarbio) were fixed with 4% paraformaldehyde (25 min, RT), permeabilized with 0.1% Triton X-100 (Biotopped), and blocked with 3% hydrogen peroxide (10 min, RT). After incubation with goat serum (1:9, Boster-Bio) for 20 min, primary antibodies against Col I, Col II, and Col X (1:100, ABclonal) were applied overnight at 4°C. Cells were then incubated with HRP-conjugated goat anti-Rabbit IgG (1:100, Servicebio) for 30 min (RT, dark), counterstained with DAPI (Beyotime), and imaged using a Nikon E400 fluorescence microscope. Image analysis was performed with Halo software (Indica Labs). For enzyme-linked immunosorbent assay (ELISA), supernatants collected 48 h after transfection were assayed using commercial kits (Jiangsu Meimian) to quantify Col I, Col II, and Col X protein levels. Absorbance at 450 nm was measured with a microplate reader (Thermo Fisher Scientific). All experiments included technical replicates in triplicate.

### Statistical analysis

The data was analyzed using SPSS 27.0 software (IBM), and visualized using GraphPad 8.00 software, OmicShare tools ( https://www.omicshare.com/tools), and Image GP ( https://www.bic.ac.cn/ImageGP). Images were compiled using Adobe Illustrator 2020 software. Significant differences were determined using one-way analysis of variance followed by Dunn test, while other data were analyzed using independent t-tests. Results are expressed as mean±standard error [19]. Statistical significance was established at p<0.05. Furthermore, all experiments were conducted in triplicate.

## RESULTS

### Vitamin D_3_ mitigates thiram-induced tibial pathology

**Figure 1:**
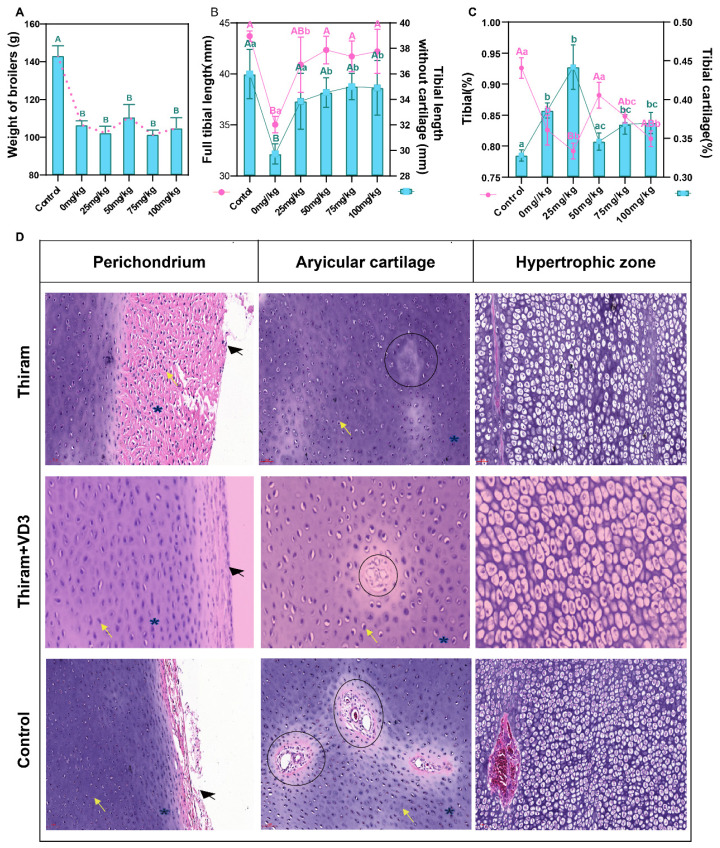
Clinical and histopathological characteristics of normal and thiram-induced TD broilers. (A) Body weight comparison (n = 6). (B) Representative tibial phenotypes and length measurements (n = 6). (C) Tibial index and proximal cartilage index (n = 6). (D) H&E-stained tibial growth plate sections (n = 3): chondrocytes (yellow arrows), perichondrium (black arrows), extracellular matrix (blue asterisks), and vasculature (circled). Scale bar: 50 μm. ^A,B^ Uppercase letters denote p<0.01; ^a–c^ lowercase letters indicate p<0.05. TD, tibial dyschondroplasia.

Exposure to thiram induced TD, indicated by a significant reduction in body weight (Figure 1A), enlarged proximal tibial cartilage (non-vascularized mass), and shortened tibial length compared to controls (Figure 1B). VD_3_ supplementation (25, 50, 75, or 100 mg/kg) attenuated cartilage damage, with 50 mg/kg VD_3_ optimal effects: increased tibial index and reduced cartilage index (Figure 1C). Histologically, thiram disrupted the organization and vascularization of chondrocytes, as evidenced by blood vessels within the articular cartilage exhibiting necroptosis or significant reduction. Additionally, hypertrophic zone chondrocytes showed excessive non-functional chondrocytes, vacuolation, shrinkage, and apoptosis. In contrast, VD_3_ partially restored the thickness of the perichondrium and the structure of the hypertrophic zone (Figure 1D). These results suggest that the broiler TD model was successfully established, providing a strong foundation for further research.

### Transcriptomic profiling identified differentially expressed genes associated with tibial dyschondroplasia

**Figure 2:**
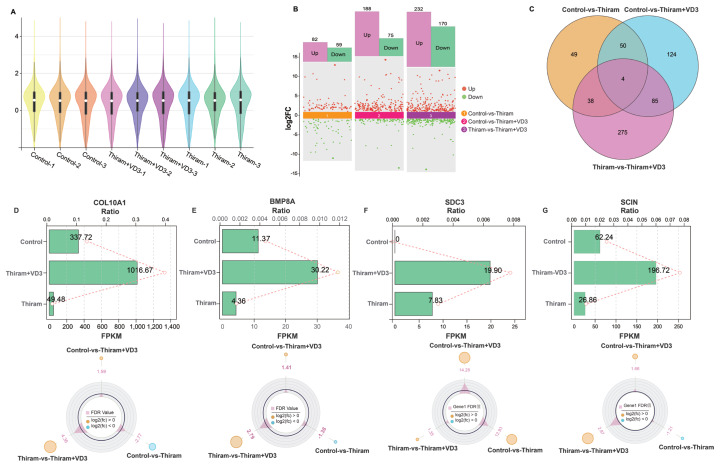
Gene expression analyses of TD, saving and control broiler tibial cartilage. (A) The violin diagram shows a visualization of the abundance of expression of genes in each group. (B) DEGs between different comparison groups. (C) Venn diagrams show the intersection DEGs in three comparisons. (D–G) The FPKM value of COL10A1, BMP8A, SDC3, and SCIN. Significant difference is considered with |fold change|≥2 and p<0.05 as n = 3. TD, tibial dyschondroplasia; DEGs, differentially expressed genes.

We employed a repeatability scatter plot (Supplement 4) to demonstrate inter-sample consistency and a violin plot (Figure 2A) to visualize the distribution of gene abundance. DEGs across the three comparison groups (Control vs. Thiram, Control vs. Thiram+VD_3_, Thiram vs. Thiram+VD_3_) were identified using the thresholds of |fold change|≥2 and p<0.05, as summarized in Figure 2B. Key genes including *COL10A1*, *BMP8A*, *SDC3*, and *SCIN* showed significant differential expressions in all three comparisons (Figures 2C–2G). In addition, *SOX9*, *TGF-β1*, and *HSPB1* displayed dynamic expression changes across specific pairwise comparisons, suggesting their potential roles in TD progression (Supplement 5).

### Pathway enrichment analysis reveals transforming growth factor-β signaling and matrix regulation

**Figure 3:**
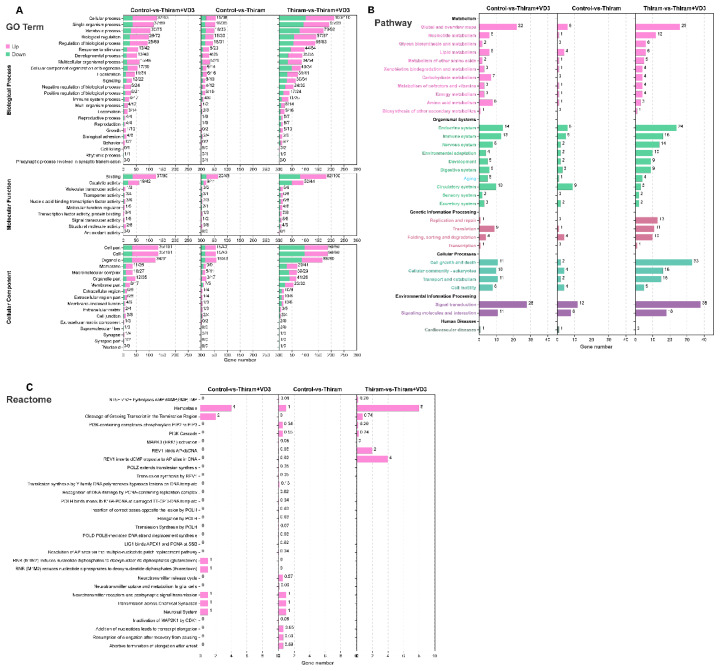
The functional predictions of differentially expressed genes (DEGs). (A) GO terms within DEGs compared to the entire genomic background. KEGG (B) and Reactome (C) pathways were significantly enriched among DEGs compared to the entire genomic background.

To elucidate the biological functions of DEGs, we analyzed the GO, KEGG, and Reactome databases for the three comparison groups. GO analysis revealed predominant associations with fundamental cellular processes, particularly in categories of cellular processes, binding, and cell part (Figure 3A and Supplement 6). The KEGG pathway mapped several major functional domains like metabolism, organismal systems, genetic information processing, cellular process, environmental information processing, and human diseases (Figure 3B). Specifically, pathways such as osteoclast differentiation, TGF-beta signaling pathway (TGF-β1, BMP8A), cell cycle, and DNA replication were enriched (Supplements 7A–7C). Pathway crosstalk analysis further revealed intricate interactions between these biological processes (Supplements 7D–7F). Notably, the Reactome results identified pathways such as hemostasis and cleavage of growing transcript in the termination region, with the latter pathway potentially involved in cartilage development and calcification (Figure 3C). Also, pathways such as striated muscle contraction, ATP hydrolysis by myosin, and calcium binding to troponin-C, were predominantly enriched in the Control vs. Thiram and Control vs. Thiram+VD3 comparison groups (Supplements 8A, 8B). Whereas cell cycle, mitotic, and S phase, were significantly enriched in the Thiram+VD_3_ group (Supplement 8C).

Focusing on four pivotal genes (*COL10A1*, *BMP8A*, *SDC3*, *SCIN*), multi-database analysis revealed their involvement-GO in positive regulation of cell differentiation, regulation of protein complex disassembly, and regulation of hematopoiesis (Supplement 9A). Reactome identified associations with collagen type X/II fibrils organization and MMP-mediated gelatin degradation (Supplement 9B). Additionally, KEGG enrichment connected these four DEGs to TGF-beta signaling (BMP8A), Fc, gamma R-mediated phagocytosis (SCIN), cell adhesion molecules (SDC3), hippo signaling (BMP8A), regulation of actin cytoskeleton (SCIN), and thermogenesis (BMP8A) (Supplement 9C). These integrated pathway data underscore a complex regulatory network that may regulate TD pathogenesis, with particular emphasis on matrix biology, differentiation control, and pathway crosstalk.

### BMP8A regulates the proliferation and apoptosis of chicken chondrocytes

**Figure 4:**
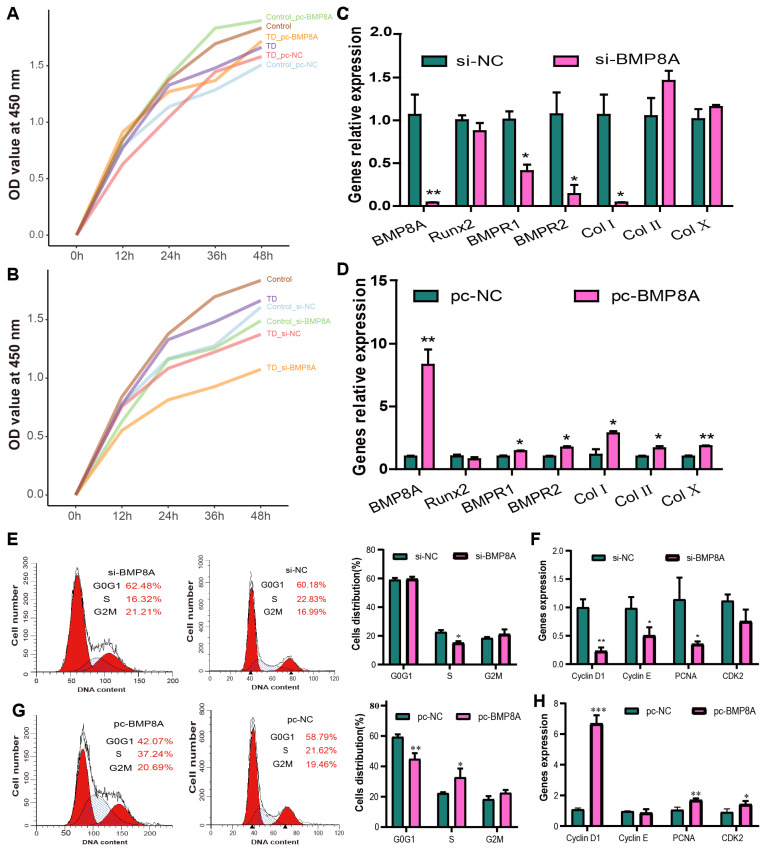
BMP8A promoted chondrocytes proliferation in the TD broiler chickens. Knockdown (A) and overexpression (B) of BMP8A respectively suppressed and enhanced chondrocyte viability. Osteogenic markers expression level of BMP8A, Runx2, BMPR1, BMPR2, Col I, Col II, and Col X after knockdown (C) and overexpression (D) of BMP8A. Cell cycle analysis in TD chondrocytes after knockdown (E) and overexpression (G) of BMP8A. Cyclin-related genes (Cyclin D1, Cyclin E, PCNA, CDK2) expression level after knockdown (F) and overexpression (H) of BMP8A. All data was presented as mean±SEM for n = 3 (Values represent the mean of three technical replicates), * p<0.05, ** p<0.01. TD, tibial dyschondroplasia; SEM, standard error of the mean.

The 4 co-differentially expressed genes are implicated in chondrocytes development. RT-qPCR revealed significant down-regulation of *BMP8A* and *Col10A1* in TD chondrocytes on days 7 and 10 compared to controls, while *SDC3* and *SCIN* expression increased on day 10 (Supplements 10A–10D). Also, functional studies using BMP8A.1227 and pcDNA3.1-BMP8A (Supplements 10E, 10F) demonstrated that BMP8A overexpression enhanced chondrocyte viability (Figures 4A, 4B), as evidenced by increased EdU+ cells (Supplement 11) and the proportion of cells in S-phase, accompanied by upregulated expression of *Cyclin D1*, *PCNA*, and *CDK2* (Figures 4G, 4H). Conversely, BMP8A silencing reduced proliferation markers and S-phase proportion (Figures 4E, 4F). BMP8A overexpression in TD chondrocytes promoted chondrogenic factors, such as *BMPR1*, *BMPR2*, *Col I*, *Col II*, and *Col X*, while knockdown suppressed *BMPR1*, *BMPR2*, and *Col I* (Figures 4C, 4D). BMP8A also showed similar effects in the control group of chondrocytes (Supplement 12). Apoptosis analysis showed that si-BMP8A elevated *Bax* and late apoptosis in TD chondrocytes (Supplements 13E, 13F), whereas BMP8A overexpression attenuated apoptosis markers like *Bax* and *Caspase-9* and early apoptosis and necrosis (Supplements 13C, 13D, 13G, 13H). Furthermore, matrix secretion assays indicated BMP8A knockdown increased Col X in control group but reduced Col II, and Col X in TD chondrocytes, with overexpression restoring Col II, and Col X secretion in TD models (Supplement 14). These findings implicate BMP8A in modulating chondrocyte proliferation, apoptosis, and matrix homeostasis, particularly in TD pathogenesis. However, the mechanistical pathways require further elucidation.

### Transforming growth factor-β1 regulates chondrocyte progress in tibial dyschondroplasia pathogenesis

**Figure 5:**
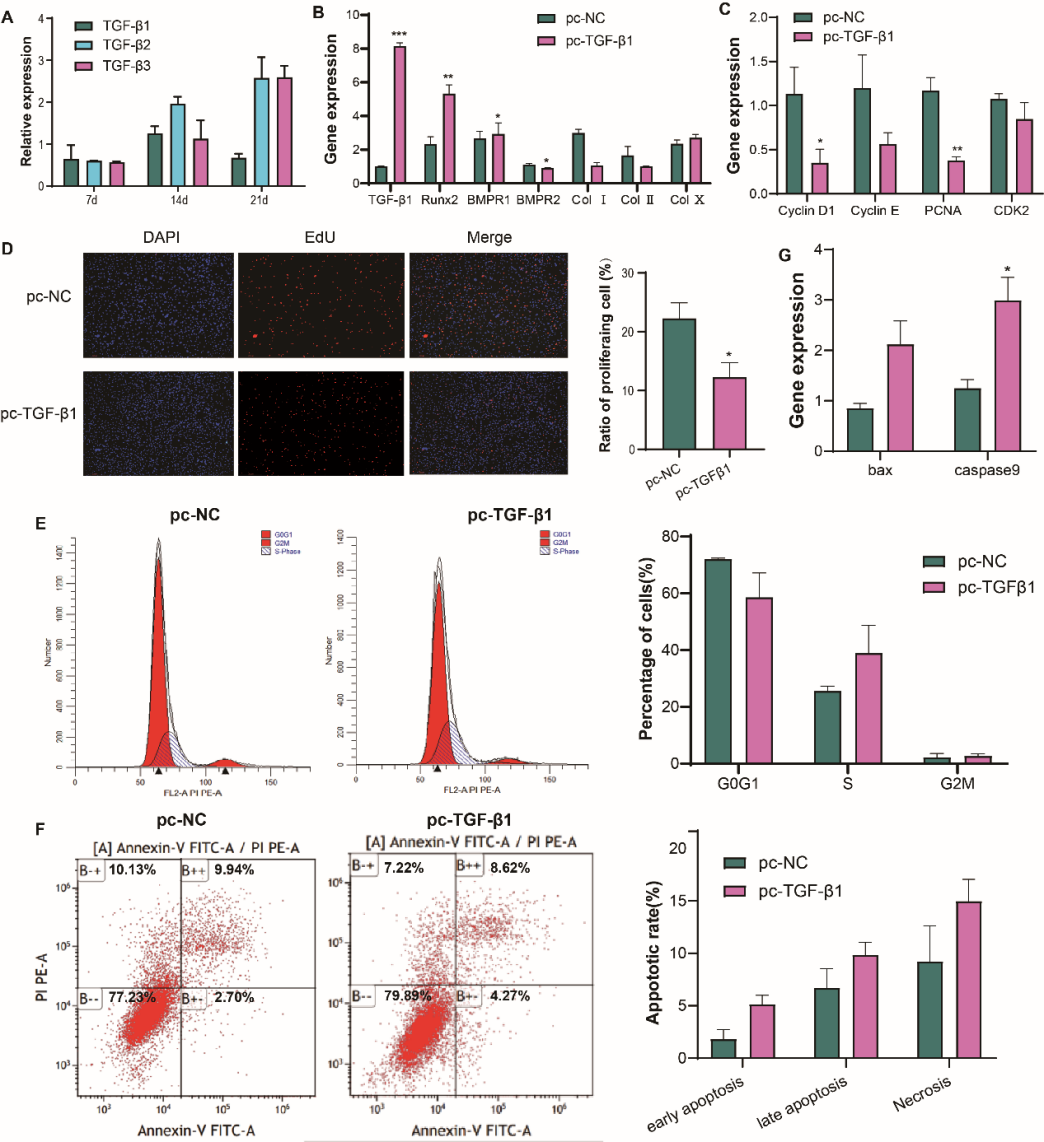
Effect of TGF-β1 on the proliferation, differentiation, and apoptosis of TD chicken chondrocytes. (A) Temporal expression profiles of TGF-β isoforms during TD progression. (B, C) The expression levels of proliferation-related genes and differentiation-related genes after transfection with pc-TGF-β1 and pc-NC. (D) EdU assays were conducted on chondrocytes transfected with pc-TGF-β1 and pc-NC for 48 hours. The red EdU fluorescence indicates proliferating cells, whereas the blue DAPI fluorescence marks cell nuclei. Images were captured at 100× magnification, with quantified EdU labeling results shown on the right. (E) Cell cycle analysis of chondrocytes at 48 h after being overexpressed TGF-β1. (F) Apoptosis analysis at 48 h after overexpressed TGF-β1. (G) Apoptosis-related genes expression detected by RT-qPCR after being overexpressed TGF-β1. Data represents SEM (n = 3, n represent the mean of three technical replicates); * p<0.05; ** p<0.01; *** p<0.001. TGF-β1, transforming growth factor-β1; TD, tibial dyschondroplasia; RT-qPCR, quantitative real-time polymerase chain reaction; SEM, standard error of the mean.

**Figure 6:**
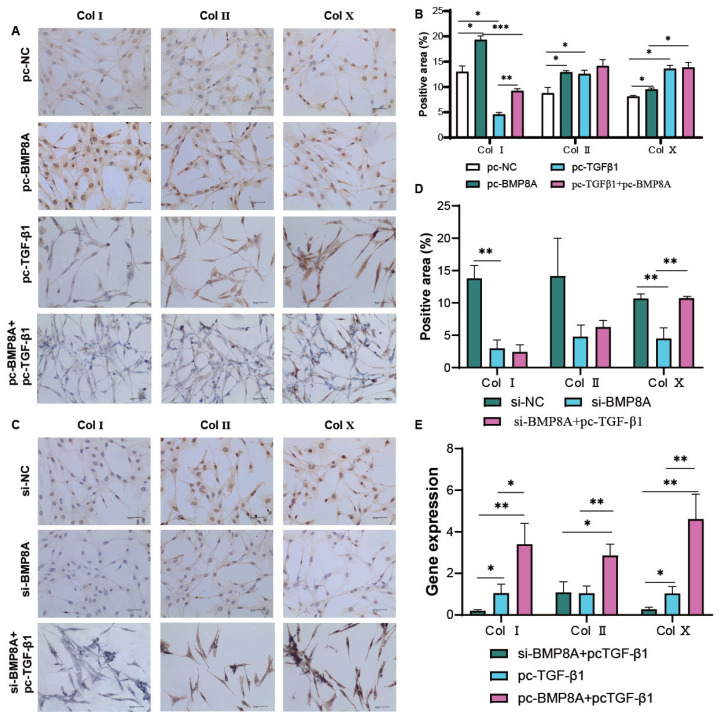
Impact of BMP8A and TGF-β1 on TD chicken chondrocyte proteins. (A, B) IHC analyzed Col I, Col II, and Col X levels 48 hours after introducing pc-NC, pc-BMP8A, pc-TGF-β1, and pc-BMP8A+pc-TGF-β1. (C, D) IHC assessed Col I, Col II, and Col X levels 48 hours after introducing si-NC, si-BMP8A, and si-BMP8A+pc-TGF-β1. Positive areas (%) are presented as mean±SD from three experiments. Bar = 100 μm, 40×. (E) Expression of the Col I, Col II, and Col X genes after co-transfection. n = 3, n represent the mean of three technical replicates, * p<0.05, ** p<0.01, *** p<0.001. TD, tibial dyschondroplasia; SD, standard deviation.

Transcriptional profiling revealed the expression of *TGF-β1* alongside *BMP8A* in the TGF-β signaling pathway during the progression of TD (Supplement 7B). While *TGF-β2* and *TGF-β3* remained stable, TGF-β1 showed biphasic expression during TD induction (Figure 5A). Consequently, we found that transfection with pc-TGF-β1 suppressed Col I while promoting Col II and Col X levels in TD chondrocytes compared to the control group (pc-NC, si-NC, Figure 6A). Cell cycle analysis demonstrated that accumulation of the G0/G1 phase and reduction of the S-phase (Figure 5E), corroborated by reduction in EdU+ cells (Figure 5D). Additionally, there was a downregulation of proliferation-related factors, including *Cyclin D1*, *PCNA*, and *BMPR2*, and an upregulation of differentiation-related factors, such as *Runx2* and *BMPR1* (Figures 5B, 5C). Despite an increase in Caspase-9 mRNA level, apoptosis, late apoptosis, or necrosis rates remained unchanged (Figures 5F, 5G). These data suggesting that TGF-β1 is a key modulator of ECM remodeling in TD chondrocytes through regulation of the balance of proliferation-differentiation.

### Transforming growth factor-β1 regulated Col I/II/X accumulation by inhibiting BMP8A

Compared to the pc-NC group, the protein-level analysis showed TGF-β1 and BMP8A differentially regulate collagen isoforms: pc-TGF-β1 increased Col II but decreased Col I, contrasting with the pro- Col I effect of BMP8A (Figures 6A, 6B). Co-transfection studies revealed that functional antagonism-BMP8A co-expression reversed TGF-β1 mediated Col I and Col X suppression (p<0.001 vs pc-TGF-β1 alone). Also, pc-BMP8A and pc-TGF-β1 significantly increased *Col I*, *Col II*, and *Col X* levels. Conversely, si-BMP8A synergized with pc-TGF-β1 to further reduce *Col I* and *Col X* mRNA (Figures 6C–6E). Intriguingly, while single transfection of either factor upregulated all collagen isoforms, their combined manipulation produced divergent effects, suggesting context-dependent regulatory interplay. This complex reciprocity highlights TGF-β1-BMP8A axis as a critical network node in TD matrix homeostasis.

## DISCUSSION

Thiram is essential for managing diseases and pests in agriculture. However, its use involves risks of bioaccumulation and environmental contamination that could harm humans and non-target species. Studies in poultry have shown that exposure to thiram or similar dithiocarbamate compounds within 24 to 48 hours can increase the frequency and severity of TD [20]. This disorder impairs the development of growth plates in chickens, leading to skeletal deformities, growth retardation, economic losses, and compromised animal welfare [4]. As well as thiram significantly reduces the weight, length, and width of the tibia while increasing the width of the tibial growth plate in broiler chicks [1]. VD_3_ and its metabolites are essential in maintaining calcium and phosphorus homeostasis. Dietary supplementation with these nutrients in broilers has consistently demonstrated positive outcomes, including enhanced growth performance, increased weight gain, prevention of rickets, and a reduced occurrence of TD [21]. Despite the 25 mg/kg, 50 mg/kg, 75 mg/kg, and 100 mg/kg concentrations of thiram that didn’t show an impact on chicks’ weight, reports indicated that they improve tibial length (or exclude cartilage) and tibial index. The 50 mg/kg dose produced measurements of the tibial index and the tibial cartilage index in TD-affected broiler chickens that were close to those of the control group. This similarity led to the selection of 50 mg/kg as the optimal experimental concentration to maximize therapeutic efficacy while minimizing adverse effects.

The development of long bones in animals is closely related to vascular development, chondrocyte functions, and matrix dynamics, all regulated by specific gene expressions. Following the establishment of a TD chicken model, we performed mRNA-seq in cartilage tissues treated with 50 mg/kg VD_3_. By comparing these results with prior data [5], we analyzed differentially expressed and co-expressed genes across control, thiram, and thiram+VD_3_ groups, deepening our understanding of the genetic factors that influence the progression of TD and treatment responses. In the present study, we identified 625 differentially expressed mRNAs, including *TGF-β1*, *SLC38A4*, and *TNN*, which are known to play a role in the regulation of growth plate cartilage development [22,23]. TGF*-β1* was also enriched in key pathways that regulate chondrocyte proliferation and differentiation, such as the TGF-*β* signaling pathway and the Hippo signaling pathway [24,25]. Additionally, *HSPB1*, *BMP2*, *COL10A1*, *Sox9*, *SDC3*, and *SCIN* are involved in regulating bone development [26,27].

*In vivo*, gene regulation is primarily regulated by the cross-talk of various signaling pathways. This study highlighted the importance of the arginine and proline metabolism pathway (ko00330) in the regulation of cartilage development. Rucci et al [28] showed that rational arginine and proline metabolism could regulate osteoclast activity by regulating chondroitin sulfate and adnexin 2, thus maintaining bone homeostasis. Additionally, the calcium-phosphorus components of the chondrocyte matrix, essential for bone development and formation, participate in multiple signaling pathways, including Ca2+/CaMKII/Nrf2 and FAK/p38 [29,30]. There are still some studies that have highlighted the significant contributions of the TGF-β and BMP superfamilies to bone formation [7,8]. Further studies are needed to explore how an interaction network of the KEGG signaling pathway centered on ko00250, ko00590, and ko04810 could regulate development and calcification of the tibial cartilage. These findings support our sequencing results and emphasize the complexity of signaling networks in skeletal biology.

BMP, a crucial member of the TGF-β superfamily, has been extensively studied for its function in regulating osteoarthritis in mice, as well as proliferation and apoptosis of TD broiler chondrocytes treated with 100 mg/kg thiram, specifically involving its ligands BMP5/6/7-related peptides [9,31]. BMP8A is involved in various biological processes such as antiviral immunity [32], and plays a key role in bone formation associated with ankylost spondylitis [33]. However, the specific functions of BMP8A in bone formation, particularly in the development of broiler chicken growth plate chondrocytes, remain poorly understood.

The TGF-β signaling pathway directly regulates endochondral ossification and bone homeostasis and indirectly influences bone formation by modulating various osteogenic factors [7,8]. For example, TGF-βR2 in hypertrophic chondrocytes is indispensable for the differentiation of terminal chondrocytes [34]. Wang et al [35] showed that transduction with hBMP2 and hTGF-β3 in BMSCs leads to more effective osteogenic differentiation than with hBMP-2 or hTGF-β3 individually. Our sequencing results showed significant changes in TGF-β1 expression, and RT-qPCR confirmed its relation to the TD process. Surprisingly, flow cytometry showed that overexpression of TGF-β1 had no significant effect on the cell cycle and apoptosis of TD chondrocytes but significantly increased the expression of the chondrocyte differentiation genes *Runx2* and *BMPR1*, suggesting involvement in chondrocyte differentiation [36]. In this study, RNA-seq and RT-qPCR analyses demonstrated a significant reduction in BMP8A expression in TD broilers. Additionally, assessments using CCK8, EdU, and cell cycle analysis revealed that BMP8A is crucial to promoting chondrocyte proliferation, especially in TD chicken chondrocytes. BMP8A was found to inhibit apoptosis of TD chicken chondrocytes, while interference with BMP8A expression did not significantly change the apoptosis rate of the healthy chondrocytes, suggesting that the effect could depend on the extent of *BMP8A* downregulation. Concurrently, significant decreases in *Runx2*, *BMPR1*, and *Col II* mRNA levels were observed, indicating an inhibition of chondrocyte differentiation [18]. The enrichment of the GO term of co-DEG *COL10A1*, *BMP8A*, *SDC3*, and *SCIN* including positive regulation of cell differentiation, regulation of cell differentiation, and cell proliferation, indirectly confirmed these results. Together, our findings demonstrate that BMP8A promotes chondrocyte progression by enhancing proliferation and differentiation.

Co-DEGs enrichment analysis including *COL10A1*, *BMP8A*, *SDC3*, and *SCIN*, using the GO and Reactome database, revealed enrichment in signaling pathways associated with protein depolymerization and collagen metabolism. Examples include cellular protein complex disassembly, formation of collagen networks, and the secretion of collagens. A decrease in Col I and an increase in Col II and Col X after transfection with pc-TGF-β1 were observed, confirming of reports of Elmallah et al [37] and Kim et al [38] that TGF-β1 promotes chondrocyte matrix production and intracellular collagen accumulation. KEGG analysis suggested that BMP8A is involved in the TGF-β signaling pathway, and the IHC and ELISA results showed that overexpression significantly increased Col II and Col X production and secretion, similar to pc-TGF-β1. This suggests that overexpression of BMP8A and TGF-β1 may contribute to intracellular production and accumulation of Col II and Col X. To verify this, we co-transfected si-BMP8A and pc-TGF-β1, and pc-BMP8A and pc-TGF-β1 in chicken chondrocytes of TD. Co-transfection of si-BMP8A further aggravated the downregulation of Col I and Col X levels caused by pc-TGF-β1, and decreased *Col I* and *Col X* mRNA levels. Conversely, co-transfection of pc-BMP8A significantly increased the protein content of Col I and the expression of *Col I*, *Col II*, and *Col X* mRNA. The observed increase in collagen production and release may be due to the dynamic balance between the anabolism and catabolism of the chondrocyte matrix [39], helping mitigate and manage excessive matrix accumulation and calcification disorders induced by thiram. In addition, it alleviates adaptive responses of chondrocytes to environmental stressors, particularly under the regulatory influences of BMP8A and TGF-β1.

## CONCLUSION

Taken together, the results obtained from this study revealed that thiram bioaccumulation can reduce the tibial index and cause deformities and abnormal apoptosis in chicken tibial chondrocytes, effects which can be mitigated by VD_3_. RNA-seq analysis identified 625 DEGs influenced by thiram and thiram+VD_3_, with BMP8A alleviating the inhibitory effects of TGF-β1 and promoting chondrocyte proliferation, differentiation, and apoptosis. Our findings improve our understanding of the genetic basis of thiram biotoxicity and suggest potential therapeutic targets for TD. However, the specific mechanisms through which BMP8A and TGF-β exert their effects require further investigation.

## SUPPLEMENTARY MATERIAL

**Supplement 1.** Chickens’ management.

**Supplement 2.** Primers used for the qRT_PCR.

**Supplement 3.** Primer sequence of siRNA of chicken BMP8A.

**Supplement 4.** Reproducibility of intragroup samples.

**Supplement 5.** DEGs of Control-vs-Thiram, Control-vs-Thiram+VD3, and Thiram-vs-Thiram+VD3 comparison groups.

**Supplement 6.** Delineation of gene ontology analysis of differentially expressed genes (DEGs).

**Supplement 7.** KEGG pathway analysis of differentially expressed genes (DEGs).

**Supplement 8.** Reactome analysis of differentially expressed genes (DEGs). **Supplement 9.** Enrichment analysis of GO (A), Reactome (B), and KEGG in *COL10A1, BMP8A, SDC3*, and *SCIN.*

**Supplement 10.** Relative expression of co-DEGs.

**Supplement 11.** Chondrocytes proliferation performed by EdU.

**Supplement 12.** BMP8A promoted chondrocytes proliferation on the control broiler chickens.

**Supplement 13.** Effect of BMP8A on chicken chondrocytes apoptosis.

**Supplement 14.** Effect of BMP8A on special protein secretion of chicken chondrocytes.
