# Supplementary material for: BMP8A, TGF-β1 regulates chicken chondrocyte proliferation, differentiation, and apoptosis induced by Thiram
Source: Anim Biosci. 2025 Sep 30;39(1):250413. doi: 10.5713/ab.25.0413 (PMC12754447; doi:10.5713/ab.25.0413)
Supplement: Supplementary file 2 [file ab-25-0413-Supplementary-2.pdf]

Supplement 2. Primers used for the qRT_PCR

Gene	Primer Sequence （5’ to 3’）
Cyclin E	F: CCAGGAAAAGAAGAACGGCAG<br>R: TCACTGCAAGCACCATCAGT
TGF-β1	F: AGCCACAGCATCTTCTTCGT<br>R: ATTGCCGTAACCCTGGTACA
TGF-β2	F: CGACATGGATCAGTTCATGC<br>R: GCTCTGTGGTTGGCTTTCTC
TGF-β3	F: CTCAGGAGAACACCGAGTCC<br>R: GGAACTCTGCTCGAAACAGG
Col I	F: GCGACAGCGGCAACATCCTC<br>R: GACCCTCTACTCCAGCACTCTCC
Col II	F: CCACCCTCAAATCCCTCAACA<br>R: GTAATCTCCGCTCTTCCACTCG
Col X	F: GCCCAGGTAACAGGGGTCTTC<br>R: GCTTGCCGATGCCAACTTCT
Runx2	F: AGGAGGGTCGCCAATAATGC<br>R: GCTTTGGGGTTCAGATTGCG
BMPR1	F: TACCTGGCATTGACCTCTCG<br>R: GCTATCGTACGTGGAGCATCA
BMPR2	F: AAGGGGCATGCGTTTCAGAT<br>R: GGTGTGGCTTGTAGGGTTCA
Bax	F: GTGATGGCATGGGACATAGCTC<br>R: TGGCGTAGACCTTGCGGATAA
Caspase-9	F: TCCCGGGCTGTTTCAACTT<br>R: CCTCATCTTGCAGCTTGTGC
Col 10 α1	F: GAACCTGGAGAAGTTGGCATCGG<br>R: CATCCCTGGCAATCCTGGCTTTC
BMP8A	F: CGGAGAGGAGGAGGAGGAGGAG<br>R: CCACCATGTTCACGAGGCTGAC
SDC3	F: AGCCAGGGGAGTTGACGACAG<br>R: CCACTGCCACCACCTCATTGC
SCIN	F: TGCCAGTGTTCTTAAGTGCCAGAC<br>R: CAACCAAACAGGCGAGGAGGATG
PCNA	F: AATGCGGATACGTTGGCTCT<br>R: CACCAATGTGGCTGAGGTCT
Cyclin D1	F: TGTCGTTCGAACCCCTCAAG<br>R: TTGCAGTAACTCGTCGGGTC
CDK2	F: TCTTCCGTATCTTCCGCACG<br>R: ATGCGCTTGTTGGGATCGTA
GADPH	F: GGTGGCCATCAATGATCCCT<br>R: CCGTTCTCAGCCTTGACAGT
