# Supplementary material for: BMP8A, TGF-β1 regulates chicken chondrocyte proliferation, differentiation, and apoptosis induced by Thiram
Source: Anim Biosci. 2025 Sep 30;39(1):250413. doi: 10.5713/ab.25.0413 (PMC12754447; doi:10.5713/ab.25.0413)
Supplement: Supplementary file 3 [file ab-25-0413-Supplementary-3.pdf]

Supplement 3. Primer sequence of siRNA of chicken BMP8A

siRNA_ID		Sequence
si-BMP8A.546	Sense	CCUGCACGUCAGCAUCUAUTT
	Antisence	AUAGAUGCUACGUGCAGGTT
si-BMP8A.884	Sense	CCAAGAAGAGCAACGACCUTT
	Antisence	AGGUGGUUGCUCUUCUUGGTT
si-BMP8A.548	Sense	CCUCAAGAAGCACCGCAAUTT
	Antisence	AUUGCGGUGCUUCUUGAGGTT
Negative control	Sense	UUCUCCGAACGUGUCACGUTT
	Antisence	ACGUGACACGUUCGGAGAATT
