# Supplementary material for: BMP8A, TGF-β1 regulates chicken chondrocyte proliferation, differentiation, and apoptosis induced by Thiram
Source: Anim Biosci. 2025 Sep 30;39(1):250413. doi: 10.5713/ab.25.0413 (PMC12754447; doi:10.5713/ab.25.0413)
Supplement: Supplementary file 4 [file ab-25-0413-Supplementary-4.pdf]

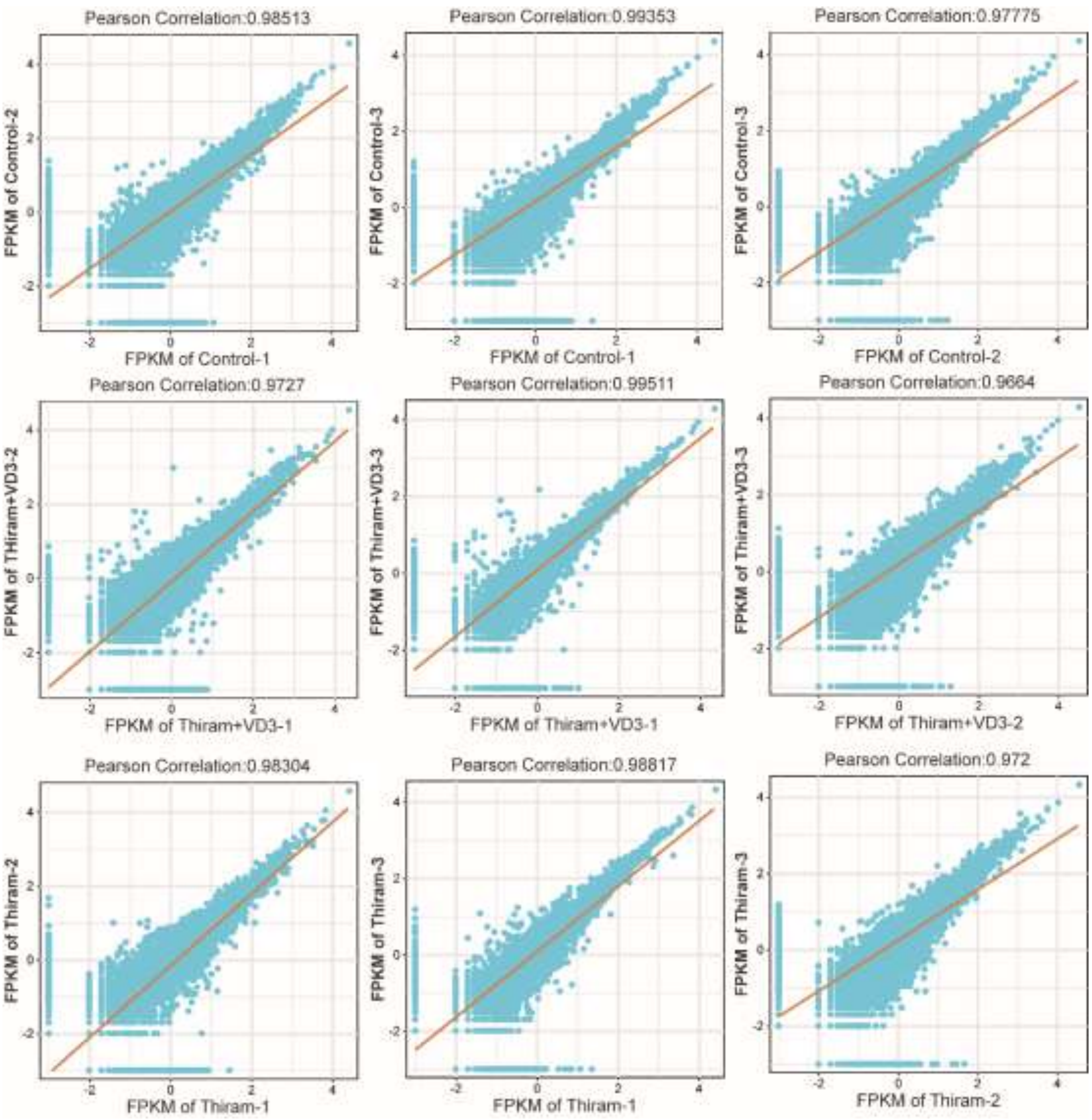

Supplement 4. Reproducibility of intragroup samples. The pairwise correlation coefficient was computed using the FPKM values of genes within the samples to evaluate the consistency within the group. Subsequently, a logarithmic transformation was applied to the FPKM values prior to visualization (n=3).
