# Supplementary material for: BMP8A, TGF-β1 regulates chicken chondrocyte proliferation, differentiation, and apoptosis induced by Thiram
Source: Anim Biosci. 2025 Sep 30;39(1):250413. doi: 10.5713/ab.25.0413 (PMC12754447; doi:10.5713/ab.25.0413)
Supplement: Supplementary file 5 [file ab-25-0413-Supplementary-5.pdf]

Supplement 5. DEGs of Control-vs-Thiram, Control-vs-Thiram+VD3, and Thiram-vs-Thiram+VD3 comparison groups.

NCBI_ID	Symbol	Control-vs-Thiram-log2fc	Control-vs-Thiram-pvalue	Control-vs-Thiram-fdr	Control-vs-Thiram+VD3-	Control-vs-Thiram+VD3-pvalue	Control-vs-Thiram+VD3-fdr	Thiram-vs-Thiram+VD3-	Thiram-vs-Thiram+VD3-pvalue	Thiram-vs-Thiram+VD3-fdr
MSTRG.28	TTN	1,660930772	1.973823698499	0,00334755	1,985615171	1.04245650631923e-05	0,00142784	0,324684399	0,366282074	0,804876641
MSTRG.31	--	-0,510961919	0,564651861	0,99993434	1,764541839	0,026282159	0,313597198	2,275503758	0,000586658	0,023021626
MSTRG.46	--	-3,29211244	0,002203674	0,13018348	0,872337431	0,383778592	0,849975379	4,164449871	0,000201746	0,010558043
ncbi_10085	CPLX2	-0,388819979	0,467800177	0,99993434	1,413285593	0,005564779	0,14098513	1,802105571	0,000472252	0,019402053
ncbi_10085	LOC1008	-3,445756682	2.561192261769	1.078922895	-1,654040883	0,008456664	0,178199799	1,791715798	0,057997837	0,387082733
ncbi_10085	HTRA1	0,257747344	0,480364872	0,99993434	-0,746386522	0,0135679	0,225257983	-1,004133866	0,000369747	0,016254088
ncbi_10085	LOC1008	-3	0,352889437	1	2,392317423	0,069241792	0,48658769	5,392317423	0,000916602	0,030995247
ncbi_10085	CORO6	2,077167861	0,008491533	0,31325121	3,323512604	1.58134073579481e-08	7.71366277541896	1,246344743	0,018213358	0,212188834
ncbi_10085	RPS26	0,112548638	0,94239781	0,99993434	1,18730798	0,001233921	0,053022296	1,074759341	0,000579863	0,022833958
ncbi_10085	TUBA1C	-0,090694851	0,676279042	0,99993434	-1,751945304	0,000996175	0,046162998	-1,661250453	0,001033417	0,033322579
ncbi_10085	LOC1008	-0,005538184	0,94597635	0,99993434	2,642750253	0,001038743	0,047425132	2,648288436	0,001034807	0,033322579
ncbi_10085	ADAMTS	-0,807354922	0,237914437	0,99993434	1,620882075	0,038285213	0,372267335	2,428236997	0,001265427	0,038276274
ncbi_10085	EIF4B	-0,026226539	0,828376017	0,99993434	1,120250614	0,004867582	0,131021566	1,146477154	0,000664838	0,024768049
ncbi_10085	FOXP4L	1,318075769	0,039667408	0,74752785	2,087085617	2.72818645228693e-06	0,000498235	0,769009847	0,075749678	0,435288348
ncbi_10085	LOC1008	-5,497851837	1.169564251865	6.6405824196	-1,231065296	0,420867774	0,872043864	4,266786541	0,175978475	0,625382401
ncbi_10085	COL10A1	-2,770798787	1.308766346767	7.121324884	1,589970918	0,000273086	0,018899009	4,360769705	7.60041837891719e-1	1.0053419990096
ncbi_10085	HLA-F10	-4,14839184	4.215464910255	0,00655354	-1,010888316	0,142153497	0,649696327	3,137503524	0,005025438	0,095635602
ncbi_10085	HLA-F10	-7,594946589	0,068211011	1	1,649813645	0,199277097	0,721447126	9,244760234	4.48509147424546e-0	0,003232207
ncbi_10085	NPR2	0,377187378	0,326196799	0,99993434	1,366689915	0,000557469	0,03028992	0,989502536	0,014777287	0,188110869
ncbi_10085	EXTL1	-0,791606019	0,005232807	0,23619163	0,64601111	0,08783708	0,539453419	1,437617129	1.04530710511274e-0	0,001102841
ncbi_10085	PRG4	-0,909340687	0,618622145	0,99993434	1,176919608	0,583348717	0,921463149	2,086260295	4.81845721955361e-0	1.1767743298420
ncbi_10085	NCAPG2	0,256090282	0,66029244	0,99993434	-1,353785939	1.19263397920118e-06	0,000248691	-1,609876221	2.67106234513425e-0	0,000352901
ncbi_10085	SRSF7L	0,278073805	0,575213177	0,99993434	-1,148168686	0,000675945	0,035675724	-1,426242491	2.75507402743106e-0	0,002322741
ncbi_10087	TGFB1	-0,558391541	0,153369517	0,99993434	0,659279875	0,256400956	0,776567295	1,217671416	0,001248387	0,038022452
ncbi_10174	LOC1017	-2,447458977	0,152526734	0,99993434	1,685566442	0,084489263	0,531661854	4,133025419	0,001664979	0,046324358
ncbi_10174	LOC1017	2,584962501	0,099338376	0,99993434	-2,321928095	0,550262886	1	-4,906890596	0,001133415	0,03533684
ncbi_10174	NEBL1	1,574354401	0,00018221	0,0221948	2,150416843	4.37714384190913e-06	0,000710556	0,576062441	0,131262212	0,558765766
ncbi_10174	ARID5A	9,85070776	3.649611251495	4.332752121	10,6998616	2.42018761289164e-14	4.546149560273	0,849153839	0,060865489	0,394325512
ncbi_10174	ERICH5	-3,807354922	0,150174506	1	1,836501268	0,151028786	0,662842958	5,64385619	0,000221455	0,011364008
ncbi_10175	LOC1017	-0,974909019	0,057710673	0,84275363	-1,832890014	0,000713567	0,036850762	-0,857980995	0,198436851	0,653601187
ncbi_10175	LOC1017	-7,191059215	1.185045682412	3.095102313	-0,777431286	0,62246518	0,932315144	6,413627929	0,035726098	0,309157339
ncbi_10175	INTS5	3,761389068	6.539054521502	0,00959478	3,026152288	1	1	-0,73523678	0,590982846	0,910912275
ncbi_10175	CDKN1C	-0,401144596	0,274274155	0,99993434	1,283586646	0,005853879	0,145781555	1,684731241	2.0219706517104e-05	0,001834315
ncbi_10175	ZFP36L1	0,056247778	0,892633743	0,99993434	1,392258044	0,006873577	0,159120889	1,336010266	0,000913642	0,030995247
ncbi_10175	C2orf72	0,054690642	0,962620371	0,99993434	-1,726077278	0,000118501	0,010052716	-1,78076792	0,000111041	0,006479667
ncbi_10175	EHBP1L1	7,291554446	0,011629168	0,38157113	8,478432581	2.15847340669831e-05	0,002489629	1,186878135	0,381297669	0,813904701
ncbi_10175	PI3	2,321928095	0,088320932	0,99288927	5,29596786	0,000434021	0,025990419	2,974039765	1.80422098362314e-0	0,000249264
ncbi_10704	LOC1070	-0,760329587	0,532584731	0,99993434	-2,468148836	0,000384861	0,024083945	-1,707819249	0,002909368	0,067214343
ncbi_10704	LOC1070	-1,741081703	0,138335319	0,99993434	1,613451058	0,053490677	0,43641838	3,35453276	0,000847784	0,029500187
ncbi_10704	LOC1070	-5,762155503	1	1	-2,401408159	1	1	3,360747344	0,001692616	0,04699414
ncbi_10705	PDKI2	0,257797757	0,784530508	0,99993434	1,930394533	0,00015231	0,012137721	1,672596775	0,000103539	0,006123189
ncbi_10705	LOC1070	-2,243271151	0,258240568	0,99993434	-8,442943496	7.18893091436509e-08	2.5547906106212	-6,199672345	0,004659109	0,091571279

ncbi_1070	LOC1070	-0,210359866	0,672228455	0,99993434	1,525766708	0,0076413	0,168899949	1,736126574	7.16332483289236e-	0,004676729
ncbi_1070	RPS19	0,345541384	0,536721374	0,99993434	1,378540543	0,001240682	0,053139161	1,032999158	0,001216265	0,037216021
ncbi_1070	LOC1070	-3,169925001	0,269249123	1	3,033015057	0,012640661	0,216856306	6,202940059	4.17712308305956e-	0,003087455
ncbi_1070	LOC1070	2,07443422	4.18455010346	0,0008674	2,866715417	8.83240497431018e-12	9.6781077506003	0,792281197	0,00498203	0,095083951
ncbi_1070	LOC1070	1,225174862	0,091673252	0,99875623	-1,165059246	0,136233247	0,64021836	-2,390234108	0,001316195	0,039271437
ncbi_1070	LOC1070	-7,14974712	7.13819213508	7.170588545	-1,595158268	0,070617062	0,489134688	5,554588852	0,002705976	0,064299839
ncbi_1070	LOC1070	-6,909215649	4.81392499908	2.417886406	-0,867644808	0,503524626	0,900444156	6,04157084	0,002215094	0,056178186
ncbi_1070	LOC1070	-7,191059215	1.18504568241	3.095102313	-0,777431286	0,62246518	0,932315144	6,413627929	0,035726098	0,309157339
ncbi_1070	LOC1070	-0,574605708	0,017504569	0,4782263	-1,427564597	1.52789608498561e-06	0,000295446	-0,852958889	0,007648049	0,124829787
ncbi_1070	SERF2	-0,803035191	0,14492061	0,99993434	0,49613532	0,406921419	0,862384685	1,299170511	0,000900876	0,030779158
ncbi_1070	IRX6	-3,702532097	1.39788273218	0,00035794	0,889219569	0,056085206	0,444255643	4,591751666	7.63316379742123e-	4.7936268647805
ncbi_1070	LOC1070	1,656347134	0,081475628	0,97733181	2,91938154	0,000351996	0,022359423	1,263034406	0,085070015	0,460173651
ncbi_1070	UBALD2	-0,302134226	0,555832148	0,99993434	1,397573594	0,013207979	0,22265604	1,699707819	0,000627942	0,024003773
ncbi_1070	LOC1070	0,092060553	0,992765828	0,99993434	1,452978116	0,005331398	0,138542588	1,360917563	0,00052008	0,020974989
ncbi_1070	LOC1070	1,00775484	1	1	-1,197095203	0,52131494	0,906368633	-2,204850043	3.8017273508745e-0	0,002924084
ncbi_1070	LOC1070	-9,451211112	1.34434311765	4.179946850	-2,099535674	0,126545573	0,619970184	7,351675438	0,070346657	0,421539887
ncbi_1125	LOC1125	-6,906890596	0,015556899	0,45186225	2,818759685	0,002540447	0,08499832	9,725650281	3.21298811498382e-	1.5693661948298
ncbi_1125	LOC1125	-9,530406337	2.18863824403	3.57267835	-0,799087306	0,61642372	0,930454338	8,731319031	0,004927824	0,095011914
ncbi_1125	LOC1125	-6,434072169	8.68692654889	1.134425738	-0,519188782	0,697452835	0,94621473	5,914883386	0,005282044	0,098528432
ncbi_1125	LOC1125	-2,15277028	6.44431452713	0,00017175	-0,016197228	0,860535801	0,980426482	2,136573053	1.14571232466719e-	0,000167885
ncbi_1125	LOC1125	-4,586240919	3.19349085956	2.085189856	-1,061344987	0,525712043	0,906581516	3,524895932	0,05821976	0,387778883
ncbi_1125	AMIGO3	-1,299560282	0,146807038	0,99993434	1,584962501	0,011336637	0,203919893	2,884522783	0,000264659	0,013109425
ncbi_3738	TENM2	-0,48860496	0,451810289	0,99993434	-2,144575572	2.01302729102712e-05	0,00236333	-1,655970613	0,011414993	0,161469748
ncbi_3739	RUNX2	-0,63093793	0,046539598	0,79011686	1,38001237	0,009989073	0,193100357	2,0109503	5.6711270830866e-0	0,000661866
ncbi_3739	EGR1	1,330547805	0,012871938	0,40898939	0,018630862	0,960639009	0,997003918	-1,311916943	0,000294313	0,014012284
ncbi_3739	SMYD1	2,756300385	5.72284814298	1.91627368	2,974975976	6.42827332752682e-08	2.4111050838263	0,218675591	0,693510203	0,935366389
ncbi_3739	DLX5	-0,942694944	0,005032741	0,22979916	0,596225188	0,081352398	0,522315762	1,538920132	4.13826595122924e-	7.5799238006682
ncbi_3739	BRCA1	0,478942794	0,353046451	0,99993434	-1,309046804	0,000348976	0,022275196	-1,787989597	2.42791717033763e-	0,002148951
ncbi_3739	PCNA	0,425248542	0,259629634	0,99993434	-0,670075808	0,007104974	0,162312737	-1,09532435	0,000236486	0,01185848
ncbi_3739	CHRDL1	0,574677433	0,090522012	0,99875623	-0,88386973	0,000773906	0,038692352	-1,458547163	1.39326699973591e-	0,000199722
ncbi_3740	CDH7	-1,762960803	0,000637668	0,05407342	0,751065791	0,250117649	0,76991632	2,514026594	3.18747855402286e-	0,002610961
ncbi_3740	MYOD1	2,800691192	0,003090433	0,16714721	2,728798273	0,00111554	0,049994472	-0,071892918	0,99017028	0,998149556
ncbi_3740	MYH1B	2,171024407	2.79170605679	0,00455711	1,622014135	0,116592941	0,604527042	-0,549010273	0,596319575	0,911551312
ncbi_3741	CDKN1B	-0,942981477	0,000302899	0,03164444	0,504423133	0,178714332	0,695241052	1,44740461	4.67256562071766e-	8.3272696494627
ncbi_3741	PTGDS	1,895180093	3.90532302729	0,00614453	2,260605277	1.0073082517435e-05	0,001394221	0,365425184	0,507664759	0,883473138
ncbi_3741	PAX3	-1,674599713	0,086384135	0,98710079	2,321928095	0,009058306	0,186786766	3,996527808	7.84632542652004e-	0,004951069
ncbi_3741	IL1RL1	0,47976192	0,407163888	0,99993434	-1,507634492	0,001719523	0,065239818	-1,987396412	2.37305327294582e-	1.8409309743299
ncbi_3741	SOX9	0,864620813	0,048162908	0,79148427	1,82039659	9.88961230880066e-07	0,0002259	0,955775777	0,003899013	0,080722421
ncbi_3741	DKK1	-2,432959407	0,456923309	1	3,078002512	0,009491207	0,190534172	5,510961919	0,000178581	0,009573702
ncbi_3741	DUSP1	1,500839263	0,000859241	0,06448751	0,235033795	0,640564196	0,936384503	-1,265805468	0,000474103	0,019417607
ncbi_3742	LEPR	-0,487805223	0,17289294	0,99993434	-1,160057008	0,000344943	0,022125169	-0,672251785	0,129954834	0,556352145
ncbi_3742	PI15	-2,175981515	3.33764691485	9.68585134	-0,901744762	0,025989548	0,312356294	1,274236753	0,003776047	0,079423463
ncbi_3742	DCX	0,422840087	0,497627326	0,99993434	-1,557068495	7.71449907676251e-05	0,007044302	-1,979908582	2.68934583904588e-	5.2157489596084
ncbi_3742	XIRP1	3,15721395	4.24789232290	3.467076615	2,760501109	2.34275703036364e-11	2.3696086301732	-0,396712841	0,435636997	0,846695924
ncbi_3787	BMP2	-1,455480542	4.62629983600	0,00012854	-0,956968516	0,020278634	0,277464891	0,498512026	0,028567207	0,275112359

ncbi_37889	FANCG	0,895976935	0,076324983	0,95279138	-0,430452552	0,392716203	0,85423227	-1,326429487	0,001734946	0,047603768
ncbi_37891	RGS2	1,04095011	0,001454502	0,09841631	1,299463531	0,00042676	0,025859289	0,258513422	0,319434414	0,773401646
ncbi_37892	BUB1B	0,314632409	0,58011582	0,99993434	-0,761429408	0,014818575	0,238111276	-1,076061818	0,000899559	0,030779158
ncbi_37892	ANKRD1	4,053731984	6.97251555196(	3.33382535	2,96907811	1.58391432760143e-08	7.7136627754189(	-1,084653874	0,144027634	0,578528355
ncbi_37892	PDE6H	-1,736965594	0,35976377	0,99993434	2,252980741	0,006459523	0,15387004	3,989946335	1.32138437467852e-(	0,001320183
ncbi_39509	PDE6B	-1,06871275	0,177070299	0,99993434	1,562419932	0,058547523	0,451519869	2,631132682	0,001330046	0,039417178
ncbi_39511	RHAG	-1,30580843	0,109683964	0,99993434	0,852193405	0,089675405	0,544889969	2,158001834	0,000727463	0,026589429
ncbi_39513	NDC80	0,408728598	0,437769641	0,99993434	-0,905410149	0,010640348	0,198210991	-1,314138747	0,001126587	0,035279794
ncbi_39516	SHISA2	-0,465577834	0,150773271	0,99993434	-1,302367723	2.50602118222776e-06	0,000470738	-0,836789889	0,029244924	0,278900728
ncbi_39519	CASQ2	2,07326896	3.88490796385(	0,00083169	1,828245248	0,000149971	0,012097934	-0,245023712	0,512180461	0,885036757
ncbi_39519	IL2RG	0,924830771	0,106621488	0,99993434	1,995554095	6.69495541648348e-05	0,006425691	1,070723324	0,033553886	0,298186417
ncbi_39521	SPP1	-1,508511599	0,060813446	0,86041473	1,830018853	0,032021381	0,34003563	3,338530452	6.22258020321832e-1	5.4708925146695
ncbi_39523	HOXA3	0,495826522	0,107226466	0,99993434	1,496920716	9.42554430840148e-06	0,001318473	1,001094194	0,012659381	0,170359094
ncbi_39526	CPZ	-0,450545073	0,085582477	0,98710079	-1,118643906	0,000297777	0,020079337	-0,668098833	0,043830586	0,342678762
ncbi_39527	MYH7B	1,41214922	0,006688407	0,27482581	2,789325838	4.13211436550614e-10	3.0185095440022	1,377176618	0,014314762	0,185417143
ncbi_39529	SOCS3	2,595360348	9.41251228228	1.755971398	0,861126178	0,149439305	0,659991173	-1,734234169	0,009319104	0,142575808
ncbi_39535	DPF3	1,049060818	0,038105184	0,73286538	0,004529658	0,174383501	0,69315461	-1,04453116	6.23969262580222e-(	0,00417711
ncbi_39537	ACLY	-0,274467481	0,218949015	0,99993434	-1,007106322	0,00012363	0,010347015	-0,732638841	0,056436025	0,380756176
ncbi_39538	SLC16A3	0,285206828	0,462556859	0,99993434	-0,794268524	0,000138516	0,011312683	-1,079475352	3.81363646616426e-(	0,002924084
ncbi_39541	SIK1	1,427441504	2.45271666515	0,00057196	0,37899828	0,264252998	0,784525327	-1,048443224	0,001151641	0,035652224
ncbi_39542	F13A1	-1,024621221	0,007034181	0,28036689	0,424887144	0,262135882	0,782498027	1,449508365	0,001598561	0,045143092
ncbi_39548	MYO6	-0,537470271	0,104900591	0,99993434	0,80098028	0,084385217	0,531661854	1,338450551	7.083761742896e-05	0,004647794
ncbi_39548	MARCO	2,082625057	0,000207573	0,02405338	1,834466768	0,001481262	0,059563059	-0,248158289	0,50222391	0,881347828
ncbi_39550	TCF7L2	-0,59644604	0,02044541	0,52602011	1,287296394	0,00251699	0,084644265	1,883742434	1.81448076412655e-(	0,000249264
ncbi_39556	WNT4	-0,861154226	0,006497561	0,27196041	0,465952184	0,267206137	0,785489267	1,327106411	2.51391656265952e-(	0,002191987
ncbi_39557	DACH2	-1,232290882	0,002126003	0,1279423	1,16525919	0,044776595	0,399435175	2,397550073	8.29196738195531e-(	0,000903756
ncbi_39561	ANGPT2	0,445450351	0,426043231	0,99993434	1,45737731	0,001151158	0,050964926	1,011926959	0,000740307	0,026895764
ncbi_39565	PHOSPH	-1,753534089	8.78419712540(	0,01220349	-0,538366512	0,172983979	0,692567212	1,215167577	0,006408718	0,110770865
ncbi_39566	IFNAR1	1,478476377	0,00581964	0,25285784	1,775932947	0,000648687	0,034673095	0,29745657	0,399338212	0,826631979
ncbi_39566	TENM1	0,787875914	0,194035564	0,99993434	-1,091147888	0,010505014	0,197293749	-1,879023802	0,000141216	0,007850273
ncbi_39570	SCD	0,487503347	0,394533132	0,99993434	-0,847006873	0,009077249	0,186786766	-1,33451022	0,000406875	0,017365285
ncbi_39570	LYG2	-2,240051088	0,095150478	0,99993434	1,15935576	0,199680556	0,721913563	3,399406848	0,001319514	0,039281617
ncbi_39571	SERPINE	-0,167109986	0,867758974	0,99993434	2,479780264	3.81465845330786e-05	0,004045076	2,64689025	1.39086282406353e-(	0,001368858
ncbi_39573	KCNAB1	0,521784312	0,330783906	0,99993434	-0,836725794	0,094241887	0,552961433	-1,358510106	0,000299254	0,014094881
ncbi_39576	TNNT3	2,850307067	0,008892499	0,32437752	4,255210189	1.0066901037398e-06	0,0002259	1,404903122	0,007347329	0,121388345
ncbi_39576	USP2	2,949016071	0,006467604	0,27196041	3,353636955	0,000466304	0,027010701	0,404620883	0,439208779	0,847444682
ncbi_39577	KRT7	1,816086208	0,000333643	0,03387981	-0,463524373	0,530122621	0,908929762	-2,279610581	0,001438695	0,041804699
ncbi_39579	LFNG	-0,715010884	0,032405074	0,68254494	0,488828652	0,289331152	0,802752013	1,203839536	4.14971239230387e-(	0,003087455
ncbi_39580	MYOM1	1,903166136	0,000183555	0,0221948	1,958902476	0,000687767	0,036029695	0,055736339	0,860031321	0,975454535
ncbi_39583	GFRA4	-0,901133325	0,000879394	0,06562289	0,21693582	0,632226156	0,935187346	1,118069145	0,000371436	0,016274083
ncbi_39584	AvBD1	-2,354430735	0,075698832	0,94870542	2,068858075	0,011196456	0,203331444	4,42328881	1.06038934670018e-(	0,000157128
ncbi_39586	HPGDS	-1,713286598	0,000380697	0,03737991	-0,986740372	0,029738951	0,329989428	0,726546226	0,055816047	0,379200891
ncbi_39587	SPI1	0,113025738	0,94663824	0,99993434	1,606035949	0,000800166	0,039554055	1,493010211	0,001364346	0,040162927
ncbi_39590	DES	2,248992289	2.39108467038	7.09663061	2,31845451	1.03981737255468e-10	8.7995249429719	0,06946222	0,734019735	0,944990275
ncbi_39593	VTN	5,049353543	2.67930591533(	1.399562237	2,971020933	0,008341226	0,17747375	-2,07833261	0,012970321	0,172780402

ncbi_3959	DIO3	0	1	1	9,067882472	0,00089379	0,042720926	9,067882472	0,00046991	0,019366161
ncbi_3959	CACNA1	1,874469118	4.63916187957	0,00707188	2,237366636	2.90791993861371e-07	7.9658831818399	0,362897518	0,137246624	0,569451121
ncbi_3959	GFRA1	0,501233151	0,282025683	0,99993434	-0,842409223	0,036348928	0,361791652	-1,343642374	0,000802198	0,028362961
ncbi_3960	XDH	0,38332864	0,839098532	0,99993434	3,447981598	0,000111835	0,009611272	3,064652958	2.31847712297244e-	0,002065951
ncbi_3960	TNNC1	2,665931342	0,000284446	0,03067595	3,762033994	9.8605226692058e-10	6.4828006288693	1,096102652	0,330274326	0,780713496
ncbi_3960	MYOM2	2,041160101	3.73050991985	1.353242473	1,277178588	0,001871395	0,069708133	-0,763981513	0,049905209	0,362816925
ncbi_3960	AGTR1	-0,106722214	0,483974101	0,99993434	1,489304641	0,001446594	0,058880837	1,596026855	0,001072936	0,034178448
ncbi_3960	MYL3	2,236437448	0,000292618	0,03106744	3,225057364	2.0940336535135e-08	9.4946374172583	0,988619917	0,124089229	0,546242278
ncbi_3960	IBSP	-1,483862531	0,006481846	0,27196041	0,763285468	0,211353284	0,733074211	2,247147999	0,000113773	0,006609883
ncbi_3960	CRYAB	2,627606838	0,000180352	0,0221948	2,655351829	1.31122505876006e-05	0,001707059	0,02774499	0,868335844	0,975516878
ncbi_3961	MST1	2,656090347	1.68060207350	5.103949413	2,59269815	1	1	-0,063392197	1	1
ncbi_3961	NTRK2	0,585897797	0,170585154	0,99993434	-0,609019768	0,139231266	0,643498037	-1,194917565	0,000222989	0,01139838
ncbi_3961	MYO1F	1,492973636	0,005627419	0,24827182	1,866652106	5.72817867051021e-05	0,005663144	0,37367847	0,399296974	0,826631979
ncbi_3961	RNASE6	1,903105648	0,002023774	0,12466259	2,262115366	3.26092102552783e-05	0,003514578	0,359009718	0,549004982	0,898297482
ncbi_3962	NOV	-0,621378148	0,051249125	0,80766771	-1,729342982	7.31760493790224e-05	0,006775999	-1,107964834	0,011655077	0,162825373
ncbi_3962	NFIX	0,129727539	0,920029737	0,99993434	-0,893700208	0,009593686	0,191185352	-1,023427747	0,001314745	0,039271437
ncbi_3962	HSPB1	1,312407648	0,013361933	0,42152826	2,048594727	3.59239159990241e-06	0,000597929	0,736187079	0,091627857	0,475556153
ncbi_3962	HRAS	-0,663219671	0,221998598	0,99993434	1,195833596	0,03820854	0,372020818	1,859053268	0,000139943	0,007820191
ncbi_3962	ETS2	-0,579517078	0,00775526	0,29699688	0,731841775	0,009480934	0,190534172	1,311358853	5.81396696188197e-	0,000672584
ncbi_3962	CDK1	0,645211917	0,143110271	0,99993434	-0,912698896	0,00844582	0,178199799	-1,557910813	3.91123570928485e-	0,002964447
ncbi_3962	CA2	-1,623783504	0,000113338	0,01495027	-0,871056088	0,034144534	0,349399585	0,752727416	0,083730214	0,457998368
ncbi_3962	AVD	3,174821534	1.61954071142	0,0028972	4,9314937	1.43262252359361e-05	0,001811303	1,756672166	0,005406399	0,099899041
ncbi_3962	ACTN2	1,833655714	3.68366142629	1.353242473	2,584116196	6.56587219402754e-10	4.5439291304877	0,750460482	0,058684457	0,389203376
ncbi_3962	COL14A	0,194346826	0,896636329	0,99993434	-2,120025049	0,001431449	0,058453816	-2,314371875	0,001512529	0,042989736
ncbi_3962	RPL27	0,036620519	0,909969744	0,99993434	1,645282267	2.45397741010352e-05	0,002757893	1,608661749	1.72334550366042e-	0,000241782
ncbi_3962	HMOX1	0,646274577	0,261404772	0,99993434	1,45333878	0,000777349	0,038717272	0,807064203	0,076013817	0,435288849
ncbi_3963	THBS4	0,307535516	0,309522978	0,99993434	-1,010631552	0,00770533	0,168899949	-1,318167068	0,00137659	0,040253807
ncbi_3963	ANK1	-0,248880312	0,376032463	0,99993434	1,176682368	0,009756283	0,191185352	1,425562679	0,001043087	0,03347015
ncbi_3963	IGFBP2	4,079727192	0,004310587	0,20591253	4,568474378	0,000950955	0,044817586	0,488747185	0,414356728	0,833008618
ncbi_3963	ALPL	-0,955088474	0,014490834	0,44218083	0,92325111	0,001068135	0,048598295	1,878339585	4.58125614571281e-	0,003283566
ncbi_3963	KTN1	-0,025216286	0,798781521	0,99993434	1,079880777	0,006030743	0,148498578	1,105097062	0,000300945	0,014124075
ncbi_3963	SDC3	12,93418229	1.28178532956	2.789805769	14,28072245	4.42888617200057e-21	5.8235424275635	1,34654016	0,000598997	0,023302589
ncbi_3963	GSTAL3	0,672958982	0,025268891	0,59444615	-0,432122092	0,124597198	0,618470575	-1,105081073	9.35978787206664e-	0,005670227
ncbi_3963	TNNI2	2,146136823	2.28322156757	0,00377425	1,856838102	0,00021382	0,015655555	-0,289298721	0,459635877	0,858101879
ncbi_3963	EXFABP	3,077803775	0,000258649	0,02838399	2,836258883	1	1	-0,241544892	0,895289593	0,979159797
ncbi_3964	TPM2	0,615954874	0,027956629	0,62837456	1,359696557	2.78286781408203e-06	0,000499357	0,743741684	0,000977921	0,032296252
ncbi_3964	TGM2	1,228189645	0,000112378	0,01495027	1,368709041	0,000414723	0,025482234	0,140519396	0,249727044	0,719083026
ncbi_3964	TNNT2	10,6794801	3.33155035139	0,00072511	10,45121111	1.25568746520672e-07	4.0950997477750	-0,228268988	0,865195955	0,975454535
ncbi_3964	TNNC2	2,411169861	5.53943212076	0,00015071	3,263655817	2.05510007424114e-15	9.0075036253989	0,852485957	0,001166091	0,035930872
ncbi_3964	TNS1	0,649462855	0,789516211	0,99993434	2,186848304	0,289394046	0,802752013	1,53738545	5.62899489061245e-	0,000661866
ncbi_3964	MYL1	2,061117769	1.56493574304	0,00038559	2,126086847	1.59438555856237e-07	4.7646762976219	0,064969078	0,562318238	0,903650605
ncbi_3964	LECT2	-1,70761826	0,00319557	0,16714721	1,312551073	0,02056199	0,279593805	3,020169334	3.27906122781954e-	0,002653022
ncbi_3964	HAPLN1	-0,800435923	0,001017756	0,0730268	0,337481347	0,513145109	0,90334303	1,13791727	0,000349134	0,015714617
ncbi_3964	HBBA	-1,625186978	0,058472311	0,84749157	1,710793478	0,052030502	0,431433982	3,335980456	6.27137489674624e-	8.2706892138289
ncbi_3964	GLUL	0,858425411	0,015643347	0,45186225	1,167830224	0,000714651	0,036850762	0,309404813	0,32521381	0,778170705

ncbi_39649	EPB41	-0,295518819	0,421111284	0,99993434	0,895957526	0,00261048	0,08646145	1,191476345	5.26006603610715e-0	0,003613008
ncbi_39649	CST3	1,040636589	0,014685478	0,44308819	-0,630781227	0,024228887	0,304866641	-1,671417816	4.03421343331123e-0	0,003022909
ncbi_39650	REL	-1,130123993	0,009674956	0,33936291	0,336427665	0,436806362	0,878963256	1,466551658	2.61258531361012e-0	0,002237323
ncbi_39651	FOS	2,085775807	0,000471517	0,04429886	-0,39405561	0,524633849	0,906581516	-2,479831417	3.96669703893548e-0	1.4314021973046
ncbi_39652	ATP1B1	-1,016665221	6.06501413195	0,0091038	0,053359978	0,927978959	0,989677978	1,070025198	0,009294722	0,142406775
ncbi_39654	ADSL	0,509536381	0,167421104	0,99993434	1,653315182	0,000767353	0,038564143	1,1437788	0,020496601	0,225798103
ncbi_39654	NACA	-0,023964738	0,793718964	0,99993434	1,547798265	0,000246666	0,017509976	1,571763003	1.02979975743954e-0	0,001095242
ncbi_40477	MUSTN1	2,237413768	0,002102145	0,12709217	2,424740209	1.7109069878748e-05	0,002073559	0,187326441	0,695707582	0,936510319
ncbi_40777	AvBD7	-2,22667978	0,029107905	0,63993288	2,274493945	0,000863891	0,041762164	4,501173724	2.34951780794756e-0	9.6829502660038
ncbi_41483	THBS2	0,019990345	0,992213413	0,99993434	-1,242845328	7.64461101802144e-05	0,0070293	-1,262835674	0,007484235	0,122916669
ncbi_41487	PDLIM3	2,088914558	2.58837084978	0,00059301	2,659433322	5.85903772318685e-15	1.6473430767898	0,570518764	0,121541242	0,542748217
ncbi_41489	GSTA3	0,677250091	0,391949997	0,99993434	2,346044182	0,001117832	0,049994472	1,668794092	0,010674608	0,155960323
ncbi_41531	ADPGK	0,328498154	0,288563292	0,99993434	-0,698873308	0,00481617	0,13057282	-1,027371463	0,000141964	0,007850273
ncbi_41549	FANCI	0,574539341	0,134910013	0,99993434	-0,694693941	0,012886848	0,219493742	-1,269233282	4.80086272972218e-0	0,003376069
ncbi_41554	MEGF11	-0,900589078	0,009693113	0,33936291	-1,476844884	0,000444991	0,026313644	-0,576255805	0,424230711	0,83979364
ncbi_41556	KIF23	0,278412716	0,594369599	0,99993434	-0,944596637	0,000438588	0,026094968	-1,223009353	0,000175241	0,009471639
ncbi_41582	GINS2	0,720703977	0,183384855	0,99993434	-1,293160791	0,027512303	0,319537282	-2,013864768	9.42927797337305e-0	0,005678234
ncbi_41589	GNL3	1,196314982	0,097800767	0,99993434	1,583076871	0,000159486	0,012557407	0,386761889	0,426239941	0,840339659
ncbi_41589	GLT8D1	0,198187726	0,779599239	0,99993434	-1,133837539	2.33404182828112e-05	0,002645717	-1,332025265	4.42952012423566e-0	8.0022618353999
ncbi_41592	GBP4L	1,063446515	0,043871988	0,77948884	2,30134044	1.06694319914827e-06	0,000229987	1,237893925	0,004396047	0,087841004
ncbi_41593	TEX264	-0,150438954	0,412727562	0,99993434	0,896794718	0,006079582	0,149142593	1,047233672	5.1121365369187e-05	0,003548361
ncbi_41594	SEMA3G	2,438292852	0,000358224	0,03543973	1,049630768	0,206058913	0,729973187	-1,388662084	0,025370615	0,258368861
ncbi_41597	IQSEC1	-1,245010724	0,000489866	0,04569401	-0,340945321	0,207308879	0,730524358	0,904065403	0,017287202	0,205718014
ncbi_41598	SUSD3	-1,657795381	0,051812542	0,80879493	2,331661125	0,004394008	0,123591746	3,989456506	4.90036378046905e-0	0,000587509
ncbi_41602	MCM2	0,766441274	0,011354521	0,37729948	-0,977244674	0,003712185	0,10923963	-1,743685949	6.06108586219592e-0	0,000102078
ncbi_41603	CHST13	-0,815430125	0,083693238	0,97961151	-1,442338161	0,000549201	0,030177148	-0,626908036	0,25844328	0,726417302
ncbi_41608	MAGI1	0,155918143	0,999317424	0,99993434	1,427722759	6.16891118823206e-05	0,006053359	1,271804615	0,027266128	0,268476247
ncbi_41609	GXYLT2	-0,160150415	0,370124194	0,99993434	-1,150234189	6.80140376281179e-06	0,001016269	-0,990083774	0,000138464	0,007770478
ncbi_41612	PLXND1	0,947364893	0,000685965	0,05669634	1,223577652	1.22042474030433e-05	0,001604736	0,276212759	0,236852552	0,70766005
ncbi_41621	BRD8	7,094517599	0,059397201	0,85143387	9,702749879	1.83798300005801e-09	1.1508399270363	2,60823228	0,240908222	0,710836403
ncbi_41622	KIF20A	0,613880847	0,767164185	0,99993434	-1,479880455	0,428489395	0,876689456	-2,093761302	0,00070766	0,026068761
ncbi_41622	RNF44	-0,712197689	0,203889415	0,99993434	0,95238198	0,092207753	0,550858582	1,664579669	0,001615847	0,045436639
ncbi_41624	ADAM19	-1,634023319	0,008565086	0,31507455	0,975678128	0,171098949	0,6910593	2,609701447	0,000250073	0,012492271
ncbi_41629	CLK4	1,310182887	0,060498089	0,85688128	2,028951374	9.09109864445903e-06	0,001285364	0,718768486	0,127261178	0,551716111
ncbi_41629	MYOT	2	0,000196677	0,02334911	2,782307504	3.93924804049616e-06	0,000647465	0,782307504	0,127697026	0,552075013
ncbi_41634	YIPF5	0,144992503	0,994518262	0,99993434	-1,147362477	0,003514262	0,105984026	-1,29235498	0,000108395	0,006353407
ncbi_41639	EEF2KM	-11,04302728	9.03244030886	6.553035444	-0,53127463	0,363829257	0,838674818	10,51175265	1.18577854119355e-0	5.2126824670868
ncbi_41645	C14H7orf	0,257107893	0,650425352	0,99993434	-0,781283237	0,020781583	0,281128631	-1,03839113	0,001375209	0,040253807
ncbi_41645	GPR146	-0,403212064	0,102385514	0,99993434	0,723994446	0,033946406	0,34817574	1,12720651	0,000580025	0,022833958
ncbi_41665	HBM	-1,526460525	0,000302237	0,03164444	1,2966318	0,005827084	0,145389612	2,823092325	3.97194726327648e-1	8.730340846816
ncbi_41665	HBA1	-1,396618035	0,007294982	0,28437362	1,732138471	0,065589052	0,473556675	3,128756506	3.8950543491545e-19	1.7122658918883
ncbi_41665	LUC7L	-0,470418161	0,263853982	0,99993434	1,088175811	0,023764512	0,302497162	1,558593972	0,000143843	0,007871402
ncbi_41667	STRIP2	1,181606806	0,27205025	0,99993434	2,47533801	0,000188314	0,014297948	1,293731203	0,02824469	0,27402812
ncbi_41675	LOC4167	1,107171524	0,000890193	0,0660513	1,042435266	0,00047563	0,027430109	-0,064736257	0,946037855	0,989211361
ncbi_41677	CDC45	0,113946506	0,920891185	0,99993434	-1,459049195	0,000121391	0,010231853	-1,572995701	0,000556954	0,022123811

ncbi_4168:	RILPL1	1,72935241	0,412041764	0,99993434	5,420957471	4.55106884546556e-08	1.76005894850078	3,691605061	4.76060914326792e-(	8.3710551175223
ncbi_4168(	KNTC1	0,502041541	0,229363356	0,99993434	-0,840219556	0,025499558	0,311670061	-1,342261097	2.91023053990087e-(	0,000372623
ncbi_4169(	MYO18B	1,919829651	0,000286581	0,03067595	2,477047162	2.99837372034513e-08	1.2320505015255	0,55721751	0,071794679	0,423906526
ncbi_4169:	DDT	0,226617007	0,589686705	0,99993434	-0,859039077	0,000859891	0,041722177	-1,085656085	0,000437315	0,018250989
ncbi_4169:	CIT	0,54030522	0,128077563	0,99993434	-1,054339834	0,000456194	0,026660005	-1,594645054	1.81939888220969e-1	1.0432274981991
ncbi_4169:	HSPB8	1,54485574	0,025435104	0,59444615	2,043068722	0,000161167	0,012600538	0,498212982	0,2911454	0,75390252
ncbi_4170:	EWSR1	0,4279073	0,432003756	0,99993434	1,21350059	0,000131643	0,010886645	0,785593291	0,029236082	0,278900728
ncbi_4170:	KIFC1	0,139099646	0,998117916	0,99993434	-1,980371193	4.59132400663008e-05	0,004753647	-2,119470839	1.07868479519362e-(	4.9054120962115
ncbi_4170:	IL4I1	3,700439718	0,240826836	1	6,851749041	5.32768602119953e-05	0,005430523	3,151309323	0,011274569	0,160918841
ncbi_4170:	MHCIA9	-3,251783271	2.35998073226	0,00056035	-0,691482824	0,236556391	0,760299064	2,560300446	9.06496387683359e-(	0,005534664
ncbi_4171	LOC4171	0,329307625	0,501251584	0,99993434	-1,555009279	0,001583087	0,062323402	-1,884316904	0,000118994	0,006880533
ncbi_4172:	CLIC3	0,733354341	0,129926831	0,99993434	1,796364138	0,000913372	0,043201165	1,063009798	0,031807267	0,290695934
ncbi_4173	MYH1C	1,760018469	2.71946018403:	1.775671527	1,367045776	1.64091207125122e-05	0,002035505	-0,392972692	0,22921992	0,698028922
ncbi_4174:	KPNA2	0,244823699	0,84078387	0,99993434	-1,033892329	0,000161951	0,012600538	-1,278716028	0,013523862	0,178352696
ncbi_4174:	NCF1	1	0,324823404	0,99993434	2,982297998	3.27609356070411e-06	0,000559446	1,982297998	0,000601966	0,023349213
ncbi_4175(	MYL10	1,756623776	0,01761029	0,4793285	3,044498124	1.49297856563457e-06	0,000293003	1,287874348	0,265480031	0,731996833
ncbi_4175:	UNC45B	2,957771765	1.05853103885	0,00027647	2,194378045	0,002364206	0,08137944	-0,763393719	0,119056035	0,53564393
ncbi_4175:	CCL26	2,206450877	0,007056651	0,28036689	3,932797725	4.46430713951532e-07	0,000119798	1,726346847	0,00734752	0,121388345
ncbi_4175:	SLC43A2	0,483113834	0,218166568	0,99993434	1,010902627	0,000768409	0,038564143	0,527788792	0,076869585	0,437907596
ncbi_4175:	RPL23A	0,16416589	0,897983812	0,99993434	1,267438855	0,001014185	0,046791296	1,103272966	0,002084303	0,053792143
ncbi_4175:	MYO18A	-0,807354922	0,002488865	0,142553	0,841090158	0,013905738	0,228272848	1,648445081	2.13732314395091e-(	5.8096460603826
ncbi_4175:	NSRP1	0,036635002	0,897233564	0,99993434	1,223950576	0,004374033	0,123420953	1,187315574	0,001766535	0,048134428
ncbi_4177(	CBLL1	0,558132234	0,193242239	0,99993434	1,231426233	0,000329114	0,021856129	0,673293999	0,01270382	0,170608941
ncbi_4177:	LMOD2	2,124258103	0,009338408	0,33319747	2,716207034	0,000487081	0,027725655	0,591948931	0,368986526	0,806759053
ncbi_4177:	AASS	0,251485677	0,553052508	0,99993434	1,027605941	0,00043336	0,025990419	0,776120264	0,011420478	0,161469748
ncbi_4177:	MDFIC	0,529094551	0,511177104	0,99993434	-1,047753131	0,066556607	0,477182568	-1,576847682	0,001390978	0,040584544
ncbi_4177:	GPR85	-0,924711873	0,147493598	0,99993434	1,886132035	0,005420035	0,139123055	2,810843908	1.77736162169373e-(	0,0016624
ncbi_4177:	BMT2	-1,497119152	1.29118167056	4.11257108	0,813073687	0,136345651	0,64051767	2,310192839	1.53548304980872e-(	0,001478099
ncbi_4177:	YAF2	-0,82304602	0,007621993	0,29535788	0,639410285	0,051611506	0,429932228	1,462456305	3.35314566556105e-(	0,000417182
ncbi_4177:	TMEM11	-2,432063602	3.83659981296	0,00611002	0,098141091	0,967234379	0,997112101	2,530204693	0,000554563	0,022095417
ncbi_4178(	PRLL	-2,980371193	0,003549583	0,18036579	0,251044609	0,70608515	0,948056126	3,231415802	0,000784723	0,027970064
ncbi_4178(	SLC38A1	-0,946362036	0,002310229	0,1346843	0,334539829	0,335665419	0,825221021	1,280901865	0,000758559	0,027332984
ncbi_4178(	SLC38A4	0,524713698	0,402036304	0,99993434	1,673027976	2.74651900460683e-07	7.6838251896968(	1,148314278	0,008086188	0,130049571
ncbi_4178(	E2F7	0,655970613	0,068587538	0,91245077	-0,636563697	0,096964908	0,558202472	-1,29253431	0,000649334	0,024390987
ncbi_4179	NTN4	0,547160468	0,304042935	0,99993434	1,760404507	0,00015092	0,01210031	1,213244039	0,001025439	0,033322579
ncbi_4179	TMEM14	-0,270451877	0,424544093	0,99993434	1,244121295	0,008115469	0,174648608	1,514573173	0,000834242	0,029105779
ncbi_4179	ARHGDI	0,608575058	0,122775737	0,99993434	1,551491808	4.85552808592215e-06	0,000769221	0,94291675	0,002655197	0,063436123
ncbi_4179	HIST1H4	-0,730701168	0,466118077	0,99993434	-6,996238081	0,0110888	0,202509213	-6,265536913	1.82481781630089e-(	0,001682916
ncbi_4179:	HIST2H4	-0,730701168	0,466118077	0,99993434	-6,996238081	0,0110888	0,202509213	-6,265536913	1.82481781630089e-(	0,001682916
ncbi_4179:	GBE	0,120294234	0,969148625	0,99993434	3,113062664	0,006398434	0,15387004	2,992768431	6.1591221719695e-0:	0,004144209
ncbi_4179:	CYP2D6	0,132926097	0,842273069	0,99993434	1,232637671	4.67831813149465e-06	0,000750185	1,099711574	4.19058546792936e-(	0,003087455
ncbi_4180	TMPRSS	-4,584962501	0,080879078	1	1,502500341	0,267357544	0,785532915	6,087462841	0,000398247	0,017107745
ncbi_4180:	MB	3,06688648	0,000257088	0,02838399	2,460888151	0,006232077	0,15175107	-0,605998329	0,307361384	0,765674714
ncbi_4180:	MCM5	0,718973841	0,034087998	0,69557983	-0,817260115	0,011369555	0,203953993	-1,536233956	1.62711146678024e-(	0,001554953
ncbi_4180:	CKAP4	0,077110439	0,901980671	0,99993434	-0,967443699	0,000247688	0,017509976	-1,044554137	0,000641651	0,024259098

ncbi_41809	MYBPC1	2,713829095	5.081110577038	1.7461637638	2,156504486	6.90318412086155e-05	0,006530214	-0,55732461	0,166404138	0,61248612
ncbi_41813	DENND5	-0,83800918	0,01589588	0,4543075	1,285064042	0,011807091	0,207833257	2,123073222	1.44231947124215e-0	0,001398626
ncbi_41818	PLEKHA	-1,282369197	7.042803097709	0,01021911	-0,223145787	0,279903099	0,793883918	1,05922341	0,001705181	0,047144503
ncbi_41819	C3AR1	1,385467177	0,024620127	0,58351042	2,131654985	0,000185799	0,014203865	0,746187807	0,110574306	0,519876628
ncbi_41826	CSTA	-0,645335119	0,620885101	0,99993434	2,947620173	0,003164972	0,098416003	3,592955291	0,001050486	0,033625741
ncbi_41832	POLQ	0,235829604	0,639695916	0,99993434	-0,816835975	0,008827175	0,183573055	-1,052665579	0,001189958	0,036580815
ncbi_41836	GTPBP8	-3,137503524	0,000266861	0,0290412	-0,339692374	0,562212944	0,917871616	2,797811149	0,00165218	0,046065423
ncbi_41838	TMEM45	-0,459125446	0,024049231	0,57490125	-1,163706246	5.83328643406472e-07	0,000150396	-0,7045808	0,009297258	0,142406775
ncbi_41840	NFKBIZ	1,574994643	0,000240591	0,02708517	1,050384264	0,054643626	0,439724014	-0,52461038	0,312419511	0,770128693
ncbi_41842	LOC4184	-1,321928095	0,677574774	1	4,217230716	0,006872933	0,159120889	5,539158811	0,001120424	0,035265268
ncbi_41842	CD200	-0,816144706	0,000694243	0,05692327	0,599809081	0,316646194	0,817410739	1,415953787	0,000442464	0,018407629
ncbi_41844	RCSD1	0,708374665	0,203561273	0,99993434	1,662678451	9.78554943337844e-05	0,008635583	0,954303786	0,007367103	0,121446689
ncbi_41844	DUSP27	2,478047297	1.699708618520	0,00041105	2,269460675	5.1421560500051e-06	0,000804931	-0,208586622	0,774515077	0,957202215
ncbi_41847	ADAMTS	1,915329676	1.149506508554	0,00214449	-0,012751907	0,92495299	0,989677978	-1,928081583	2.04472225281929e-1	2.0742920823216
ncbi_41849	EVA1C	-0,860486018	0,02169042	0,5500101	0,939194976	0,02281577	0,29703422	1,799680994	6.60141102980817e-0	0,000744071
ncbi_41851	RCAN1	0,865127798	0,019519816	0,51049508	-0,154009586	0,56321748	0,918246865	-1,019137384	0,001456631	0,042127314
ncbi_41858	CYBB	0,957889163	0,0525033	0,815866	1,769018116	5.62959997412637e-05	0,005607849	0,811128953	0,096990548	0,489485304
ncbi_41860	KLHL34	2,745139526	4.946060596786	0,0009664	3,188026808	1.47144011026548e-07	4.49952697904205	0,442887282	0,376476703	0,810489428
ncbi_41860	MAP7D2	1,115477217	0,531416352	0,99993434	3,772589504	0,000391267	0,024267756	2,657112286	0,00097379	0,032267192
ncbi_41860	MAP3K1	-1,91337181	0,000335098	0,03387981	0,533143921	0,433030923	0,87742103	2,446515731	1.25925576878372e-0	0,001287369
ncbi_41861	REPS2	0,625666471	0,218104845	0,99993434	-0,996656557	0,042727612	0,392865071	-1,622323028	8.15445396193054e-0	0,005096727
ncbi_41871	MAP4K4	-0,622398307	0,02617558	0,60180792	0,657040179	0,043314434	0,394966359	1,279438486	7.71439912234944e-0	1.7243643326363
ncbi_41871	IL1R2	-0,901221846	0,229468937	0,99993434	1,424943242	0,030960344	0,33602713	2,326165088	0,000200732	0,010546801
ncbi_41874	ADPRHL	2,324234562	0,000778226	0,05978148	3,074505436	9.98016213940825e-07	0,0002259	0,750270874	0,098670927	0,491974361
ncbi_41876	ARGLU1	-0,45714153	0,301660849	0,99993434	0,844776604	0,108196252	0,584928864	1,301918134	0,000396585	0,01709205
ncbi_41881	ACOD1	1,919080005	0,072126218	0,92917414	2,939739475	0,000171588	0,013194201	1,020659471	0,316486428	0,771922716
ncbi_41882	DIAPH3	0,495923972	0,38872535	0,99993434	-1,146380537	6.22681028224933e-06	0,000941107	-1,642304509	0,000126956	0,007248043
ncbi_41883	RGCC	-1,165899847	0,00024723	0,02759471	-0,033271855	0,775158062	0,962267304	1,132627991	0,004897924	0,094573682
ncbi_41888	CKAP2	0,609530005	0,137136966	0,99993434	-0,716485047	0,019171516	0,269322937	-1,326015052	0,000364499	0,016185217
ncbi_41889	UFM1	0,266028225	0,731590576	0,99993434	-1,250372732	0,000338605	0,022125169	-1,516400958	0,001020202	0,033322579
ncbi_41893	RPL21	0,18256193	0,908658725	0,99993434	1,894181233	8.15124360450238e-06	0,00117781	1,711619303	0,000227207	0,011480492
ncbi_41894	SPATA13	-0,09169569	0,481533328	0,99993434	1,373504921	1.8173033835007e-07	5.31016048658905	1,465200612	3.43271273069181e-0	1.2934461569246
ncbi_41902	GAB2	-1,668471489	2.777174029008	1.1333473639	0,822216437	0,011033545	0,202061388	2,490687926	3.36685649047704e-1	2.4667835220228
ncbi_41907	GDPD5	-0,31267892	0,238947121	0,99993434	0,966273577	0,008441593	0,178199799	1,278952497	5.7725517887116e-07	0,000100169
ncbi_41908	RRM1	0,584435104	0,046450416	0,79011686	-0,788495895	0,003027474	0,095923521	-1,372930998	1.87971641859516e-0	0,000255564
ncbi_41911	L3MBTL	-7,845490051	2.977890140190	1.1784323430	-7,845490051	3.66938611549818e-08	1.46208357674805	0	1	1
ncbi_41917	PPP1R16	-0,268703156	0,280771923	0,99993434	1,983403489	0,000464148	0,027004797	2,252106645	2.89841806504998e-0	5.5397590495476
ncbi_41917	PLCG1	1,158545939	0,009427601	0,33455176	1,447458977	0,000339149	0,022125169	0,288913038	0,313676279	0,771281812
ncbi_41918	RBPJL	-2,116386575	1.817116252314	0,00316396	-0,340915714	0,321244673	0,818454991	1,775470861	0,0001815	0,009690764
ncbi_41919	ADA	-0,146443568	0,636479269	0,99993434	1,081223795	0,018208798	0,262530139	1,227667363	0,00146684	0,042329738
ncbi_41921	EPB42	0,445411148	0,785721354	0,99993434	2,824789515	0,004855022	0,131021566	2,379378367	0,00134431	0,039661663
ncbi_41923	MRGBP	0	1	1	10,34799039	0,000214313	0,015655555	10,34799039	7.43227638130172e-0	0,004735114
ncbi_41923	SLC17A9	0,61287198	0,052710243	0,8163318	-1,307905866	0,002095251	0,075027531	-1,920777847	2.94754502368639e-0	0,000373771
ncbi_41924	NKAIN4	0,523951505	0,186326065	0,99993434	-0,907450757	0,016977241	0,251672765	-1,431402262	2.78278034241593e-0	0,002322741

ncbi_41924	EEF1A2	1,944354242	0,000200747	0,02361765	2,39354918	1.4176873243653e-06	0,000286787	0,449194938	0,041831419	0,334956132
ncbi_41924	PPDPF	1,485146774	0,000147954	0,01857816	1,319102666	0,000846617	0,041230228	-0,166044108	0,741568903	0,947126717
ncbi_41936	RCC2	1,918470115	0,000418579	0,04019286	2,694329184	7.9398035541861e-09	4.3500198722497	0,775859069	0,114320994	0,527155688
ncbi_41940	MMP23B	-0,193951275	0,425523338	0,99993434	-1,596497831	2.55576324632523e-06	0,00047332	-1,402546555	9.22240477871099e-0	0,000139799
ncbi_41942	FAM132	1,766327654	0,000714865	0,05692327	-1,004501392	0,123776315	0,617425934	-2,770829046	2.15857337699993e-0	5.8096460603826
ncbi_41946	SPEN	-0,487697753	0,051812933	0,80879493	0,792951857	0,002502195	0,084362479	1,28064961	1.28835505347407e-0	0,001306987
ncbi_41948	VPS13D	9,351675438	1	1	9,112005026	3.99777416190755e-13	6.5708415568653e	-0,239670413	0,920572347	0,984048248
ncbi_41950	C1QA	1,710291311	0,00021638	0,0247869	2,089315831	2.74349477695771e-05	0,003006184	0,379024521	0,421098756	0,836701808
ncbi_41952	CDCA2	-0,015724352	0,659266835	0,99993434	-1,181702169	4.48889240205404e-05	0,00468448	-1,165977817	0,007200249	0,120426936
ncbi_41956	RPA2	0,592902999	0,151760571	0,99993434	-0,506035161	0,092533571	0,551052501	-1,098938161	0,000347645	0,01570115
ncbi_41957	RLF	1,644519345	0,001017066	0,0730268	2,128809148	3.80127428100937e-10	2.9401738541760	0,484289802	0,352755523	0,794016016
ncbi_41962	CSF3R	1,061927749	0,18189821	0,99993434	2,387891357	1.44897831235722e-06	0,000288676	1,325963608	0,059833345	0,391933312
ncbi_41970	CCDC15	0,586255818	0,243972872	0,99993434	-0,955409849	0,010144481	0,193100357	-1,541665667	0,000644132	0,024270889
ncbi_41973	NCAPD3	0,399862155	0,185713134	0,99993434	-1,000929272	0,000280312	0,019297471	-1,400791427	6.47476580843319e-0	0,000105419
ncbi_41975	TRIM29	-2,277984747	0,00060904	0,0526719	0,487549999	0,211283052	0,733074211	2,765534746	8.15982240772646e-0	0,000126602
ncbi_41981	KCTD20	-1,026786986	4.03220957435	0,00011447	0,031680438	0,936862018	0,99089436	1,058467424	0,000903714	0,030796331
ncbi_41984	DYRK3	-1,275051371	0,00342101	0,17455786	0,141828544	0,927760189	0,989677978	1,416879915	0,00027055	0,013313459
ncbi_41986	IRF6	-1,169142841	0,026154421	0,60180792	-2,356769844	0,000232629	0,016806818	-1,187627003	0,153892829	0,594816899
ncbi_41991	TSPO2	-2,86644967	0,000128682	0,01663816	0,075206344	0,89721284	0,986079207	2,941656014	1.66616119741658e-0	4.6751774194744
ncbi_41991	APOBEC	1,766960334	0,001593165	0,10344872	1,759818265	0,001076528	0,04881127	-0,007142069	0,925180097	0,984767968
ncbi_41993	BTG2	1,452873411	4.65718337331	0,00707188	-0,374739766	0,304435152	0,810829804	-1,827613177	9.4764193218236e-0	2.9063957678188
ncbi_42000	RPL23	-0,39226271	0,622376245	0,99993434	2,150388131	0,03345997	0,346156195	2,542650841	1.64942418262253e-0	0,000233899
ncbi_42009	ACSBG2	0,582008253	0,089961265	0,99813438	-0,66681051	0,001468082	0,059214147	-1,248818762	6.11478925642252e-0	0,000102078
ncbi_42010	SBNO2	1,01486631	0,000324702	0,03338807	0,718508689	0,021005498	0,282125937	-0,29635762	0,469818469	0,864146825
ncbi_42011	COPE	0,246173259	0,554491734	0,99993434	-0,819493416	0,000887534	0,042591891	-1,065666675	2.83267546775374e-0	0,002349517
ncbi_42014	KLF2	0,743205697	0,22528836	0,99993434	2,381612722	1.60675425592874e-06	0,000306191	1,638407025	7.62065210959703e-0	1.7243643326363
ncbi_42015	UHRF1	0,60474938	0,054678433	0,83049047	-0,630076502	0,007607125	0,168899949	-1,234825882	8.2715771030269e-06	0,000903756
ncbi_42019	ZNF704	0,075931767	0,843575866	0,99993434	1,022480579	0,000550804	0,030177148	0,946548813	0,00028307	0,01367445
ncbi_42020	CA3A	0,957951266	0,170316946	0,99993434	2,84316834	6.60124595161204e-08	2.4111050838263e	1,885217074	0,002643376	0,063268317
ncbi_42021	ATP6V0I	-3,685050825	0,007234055	0,28284289	1,451321617	0,1597983	0,675411128	5,136372442	2.32503158550593e-0	4.6458358408563
ncbi_42023	CPQ	-0,056938042	0,756117303	0,99993434	-1,310233783	7.12721292644632e-05	0,00669398	-1,253295741	3.67288902193878e-0	0,002883218
ncbi_42028	MAL2	-0,425187618	0,333342675	0,99993434	-1,445704636	0,000761818	0,038527464	-1,020517017	0,0597978	0,391933312
ncbi_42028	COLEC1	0,552088697	0,186471677	0,99993434	-0,715567557	0,055118554	0,441174532	-1,267656254	6.89584591188266e-0	0,004547121
ncbi_42030	PSCA	-5,965784285	2.26314062452	2.955435341	2,840472389	0,018057991	0,261537128	8,806256674	2.11076841097018e-1	1.1598672418281
ncbi_42031	COL22A	-1,507880093	1.45600676800	6.556549097	0,613488915	0,130900963	0,629329711	2,121369008	4.01591456629302e-0	1.4314021973046
ncbi_42033	MTSS1	-0,55507429	0,043145715	0,77502048	0,704151856	0,044531699	0,398439136	1,259226146	7.01541798502145e-0	0,000112828
ncbi_42034	FBXO32	0,42637059	0,372580378	0,99993434	1,656723727	2.86358365994061e-06	0,000502043	1,230353137	0,000367954	0,016229366
ncbi_42036	HSF1	0,699363267	0,103051016	0,99993434	1,440572591	1.42478111658211e-05	0,001811303	0,741209324	0,029926189	0,282106208
ncbi_42036	RARRES	-0,171789772	0,553553673	0,99993434	1,22635543	0,004279118	0,121788136	1,398145202	9.64118580758371e-0	0,000144486
ncbi_42038	KLHL40	3,142957954	1.56214893801	0,00283335	2,958420896	2.57367383932343e-05	0,002867901	-0,184537058	0,79230137	0,961061197
ncbi_42039	HHATL	1,709813988	0,000564598	0,04997854	2,377100214	5.41056385026427e-05	0,005472577	0,667286226	0,47846118	0,868768635
ncbi_42039	OBSCN	1,605947526	2.31043867859	2.011467913	1,835020821	2.59158510339448e-08	1.1331383099956	0,229073295	0,40180063	0,827909928
ncbi_42043	EXOG	-0,143194636	0,544184826	0,99993434	1,109347396	0,000484729	0,027711753	1,252542032	0,000502158	0,020439676
ncbi_42049	APBB1IP	-0,766870859	0,036551612	0,71886672	1,057839346	0,000189204	0,014297948	1,824710205	2.52640277666965e-0	0,002191987

ncbi_42052	CUBN	1,192645078	0,00798706	0,30145379	-0,342686655	0,467944889	0,889137795	-1,535331733	5.35749268627926e-	0,00364199
ncbi_42057	PDK4	1,711874613	0,000341483	0,03404143	0,829316444	0,00674918	0,157994842	-0,882558169	0,067759482	0,41351784
ncbi_42057	C1GALT	0,610029483	0,051260814	0,80766771	-0,438188464	0,06721717	0,479837176	-1,048217947	0,000136626	0,007700122
ncbi_42058	SCIN	-1,212280991	0,000225423	0,02559825	1,660127965	0,000171481	0,013194201	2,872408956	3.1526685929601e-0	1.2228645118811
ncbi_42073	AOAH	-0,700439718	0,519590935	0,99993434	1,783376059	0,053189409	0,435755475	2,483815777	0,000275557	0,013459433
ncbi_42073	ANLN	0,359303162	0,373297194	0,99993434	-1,08802774	0,00020044	0,014865283	-1,447330902	3.18460169490702e-	5.9997895932048
ncbi_42074	EEPD1	-0,229708335	0,379882259	0,99993434	0,777972402	0,02870131	0,326182818	1,007680737	8.93245149306928e-	0,005504728
ncbi_42076	CDK13	-0,226068079	0,555202393	0,99993434	1,466422886	0,006938626	0,159782822	1,692490965	0,000142862	0,007850273
ncbi_42078	CUL1	0,153011619	0,815728882	0,99993434	1,205357512	0,001712954	0,065239818	1,052345894	0,000627099	0,024003773
ncbi_42079	TRIP13	0,311822378	0,591874628	0,99993434	-1,255610198	0,004428643	0,124162529	-1,567432576	0,00022587	0,011456836
ncbi_42085	MAK	-3,983511877	1.93844859013	0,00333082	-0,888354644	0,344585983	0,829238853	3,095157233	0,040721442	0,33170746
ncbi_42098	MURC	1,52905376	0,046722084	0,79011686	2,493539473	0,000293113	0,01986671	0,964485713	0,099811472	0,494810547
ncbi_42099	NR4A3	2,239700997	7.66609849103	5.888916482	0,102197473	0,947114367	0,993114524	-2,137503524	4.19685495827517e-	1.4565295576245
ncbi_42099	SEC61B	0,121462884	0,982262359	0,99993434	-1,098777358	0,000754029	0,038280786	-1,220240242	1.77082279331858e-	0,0016624
ncbi_42103	SPIRE1	-8,544320516	0,023105384	0,56322399	2,957516669	0,091867993	0,550809987	11,50183718	5.3375369236369e-1	1.7597859237230
ncbi_42111	PCMTD1	-0,773523388	0,010024456	0,34546727	1,0411087	0,001282804	0,054153609	1,814632088	4.02325547237618e-	1.0203594840326
ncbi_42114	CHD7	1,482392767	0,500886321	0,99993434	3,97188162	0,024472095	0,307045398	2,489488852	2.16396863125217e-	0,001941389
ncbi_42116	TNNI1	2,092164522	0,0005481	0,04948319	2,750706038	9.52395270421317e-08	3.2110372848128	0,658541516	0,234602592	0,705412444
ncbi_42116	TUBA3E	0,722243524	0,018140794	0,4854521	-0,592692229	0,029230334	0,32766382	-1,314935752	3.38179760054406e-	0,002702979
ncbi_42117	SKIV2L	9,252665432	7.14810551202	3.33382535	8,395177077	0,060428574	0,459164915	-0,857488356	0,441139414	0,847444682
ncbi_42118	FKBP5	0,343295801	0,136499771	0,99993434	2,079982548	2.55372448763735e-07	7.2997659321616	1,736686747	1.34647092512998e-	0,001335132
ncbi_42120	FANCL	0,789131163	0,694746488	0,99993434	-0,435300403	0,786321825	0,965572066	-1,224431567	0,000588284	0,023021626
ncbi_42122	BUB1	0,390299385	0,421056917	0,99993434	-0,786508137	0,004803481	0,130497867	-1,176807522	0,000591784	0,023090084
ncbi_42128	LOC4212	-1,415037499	0,054066507	0,82676172	0,700439718	0,281536038	0,795427022	2,115477217	0,001474176	0,042356051
ncbi_42129	EPCAM	-8,803054785	0,000191066	0,02289106	-1,856635825	0,080339035	0,51783234	6,94641896	0,059214498	1
ncbi_42136	ATF3	1,897957471	0,000155	0,01927751	0,837569535	0,068347212	0,482651712	-1,060387936	0,045821608	0,348775979
ncbi_42137	DTL	0,636896577	0,140026476	0,99993434	-0,972272784	0,009913063	0,193100357	-1,609169361	7.32656669992452e-	0,000116413
ncbi_42139	KCNG3	2,115477217	0,138122992	0,99993434	2,613817363	0,001035604	0,047425132	0,498340146	0,571491295	0,905318899
ncbi_42142	KCNK5	-0,905428158	0,015968079	0,4543075	0,89659216	0,073563191	0,495930214	1,802020318	0,000652868	0,024390987
ncbi_42143	CCDC16	0,210642151	0,714547247	0,99993434	-0,869634113	0,011842118	0,207893205	-1,080276264	0,000947414	0,0317926
ncbi_42150	EXO1	1,010647244	0,057524834	0,84275363	-0,728087443	0,166236184	0,683382	-1,738734687	0,000194569	0,010305151
ncbi_42153	ACTA1	2,09636895	3.49000478783	1.340469780	2,450539178	3.88050148332833e-11	3.6446224288774	0,354170228	0,088422086	0,46764211
ncbi_42154	C3H1OR	-0,558524941	0,226059251	0,99993434	1,118845094	0,019721675	0,272254333	1,677370034	0,000509822	0,020624323
ncbi_42158	FNDC1	0,117953826	0,945021811	0,99993434	-1,12896657	0,00029311	0,01986671	-1,246920396	0,000119475	0,006880533
ncbi_42159	CSTF3	0,556350853	0,131904273	0,99993434	1,031004292	0,000434854	0,025990419	0,474653439	0,04632976	0,351111903
ncbi_42162	LRP11	-1,275634443	0,081284847	0,97733181	1,659659868	0,004340104	0,122726946	2,935294311	5.57590196949084e-	1.8855126967601
ncbi_42163	PPP1R14	0,070921786	0,96074587	0,99993434	-1,292409008	0,016449075	0,2484307	-1,363330794	0,000634232	0,024174123
ncbi_42163	IYD	-2,958179824	0,000549434	0,04948319	0,773624065	0,20453726	0,728810024	3,731803889	1.31291272610226e-	0,001320183
ncbi_42164	FBXO5	1,08865762	0,31664428	0,99993434	-4,827819025	0,011835852	0,207893205	-5,916476644	2.47241262879766e-	4.8665936938184
ncbi_42164	TIAM2	-0,486024959	0,062610445	0,87732517	0,805288891	0,011691659	0,206981062	1,29131385	0,000217369	0,011197913
ncbi_42167	HECA	1,060567614	0,66341417	0,99993434	1,352063813	4.06702732041789e-05	0,004278187	0,291496199	0,827747962	0,967781671
ncbi_42167	REPS1	-0,458205358	0,024394343	0,57921041	0,654062513	0,06223471	0,463521809	1,112267871	7.51436473731917e-	0,004764396
ncbi_42168	IFNGR1	0,472762756	0,238892416	0,99993434	-1,649424683	0,006067087	0,14911425	-2,122187439	0,001054649	0,033677268
ncbi_42169	PDE7B	1,159780444	0,013780816	0,42746718	1,646868406	0,000272683	0,018899009	0,487087962	0,047695212	0,354368705

ncbi_42169	RPS12	0,807056084	0,034325442	0,69604961	1,817478752	6.26413824925792e-15	1.6473430767898	1,010422668	0,001024257	0,033322579
ncbi_42171	C6orf58	-8,79224803	0,0038249	0,18991195	0,679427185	0,637820711	0,936063893	9,471675214	5.15709661987041e-0	0,003560827
ncbi_42175	SLC22A1	-2,980634675	7.270846730011	1.054999860	0,83914703	0,11061609	0,589155842	3,819781706	1.08241371830814e-1	8.9217950731548
ncbi_42176	SESN1	0,113689768	0,768926201	0,99993434	1,663117784	3.56113733106003e-12	4.6825394766108	1,549428016	4.45732282385073e-0	0,003229845
ncbi_42178	AIM1	-3,751740947	2.87388975117	0,0006361	0,024982357	0,90156067	0,986799145	3,776723303	0,000105243	0,006196175
ncbi_42181	PM20D2	-1,415037499	0,046226741	0,79011686	2,16746935	0,000671672	0,035612144	3,582506849	1.43892351012813e-0	4.1253311416456
ncbi_42182	RARS2	-1,433610884	0,016108858	0,45678892	0,435249304	0,391484004	0,853116922	1,868860188	0,00031893	0,014655216
ncbi_42183	NT5E	-1,255910922	0,000136919	0,01735941	0,880334986	0,055584309	0,442027997	2,136245908	3.29981057529196e-0	6.1292819530916
ncbi_42193	RRM2	0,74597088	0,062051795	0,87226522	-1,098018245	0,000386471	0,024083945	-1,843989125	2.92300933192143e-0	7.5585582488980
ncbi_42194	LPIN1	1,95112026	0,039668949	0,74752785	-0,730629131	0,492129403	0,897105876	-2,681749391	7.33082136271018e-0	2.301877907891e
ncbi_42200	PBK	0,571708634	0,130354981	0,99993434	-0,847822941	0,016462778	0,2484307	-1,419531576	0,000121899	0,006989593
ncbi_42204	MCM3	0,701893513	0,016997119	0,47226677	-0,961176229	0,000529282	0,029423807	-1,663069742	6.44802309838038e-0	1.5185094396685
ncbi_42204	CENPQ	0,67445364	0,228795722	0,99993434	-0,874158293	0,024961732	0,309935615	-1,548611932	0,00089678	0,030779158
ncbi_42207	CEP131	0,022827929	0,829660716	0,99993434	1,088139432	0,001510705	0,060012869	1,065311503	0,000574823	0,022765043
ncbi_42215	LOC4221	1,114004629	0,071093155	0,92562862	-0,921796093	0,159387136	0,67496987	-2,035800721	0,000224624	0,011437627
ncbi_42216	HEPH	-0,337632996	0,243423643	0,99993434	1,609367813	0,000808768	0,039680944	1,947000809	6.33218652456551e-0	0,000719904
ncbi_42217	MSN	0,37848077	0,886075176	0,99993434	-13,54954404	7.45782995596363e-09	4.2636089604767	-13,92802481	0,000818716	0,028639855
ncbi_42219	MPP1	0,575573997	0,085198769	0,98658654	1,014934262	0,000104783	0,00914706	0,439360266	0,122939828	0,545350978
ncbi_42220	GCNA	0,361608443	0,442016212	0,99993434	-0,889689855	0,02585313	0,311670061	-1,251298298	0,001017033	0,033322579
ncbi_42223	RAP2C	-3,386581053	0,377358733	0,99993434	1,105272043	0,671453089	0,942458692	4,491853096	0,000297378	0,014056698
ncbi_42226	SYTL4	2,747412382	7.673218931407	4.554753001	1,351812375	0,01040238	0,196263157	-1,395600007	0,002817427	0,065996855
ncbi_42232	UPRT	0,355999508	0,45800415	0,99993434	-0,826203823	0,010514976	0,197293749	-1,182203331	9.76677179936607e-0	0,001055772
ncbi_42234	COL4A5	0,622995633	0,255075347	0,99993434	1,366474534	0,000807188	0,039680944	0,743478901	0,061356769	0,395589006
ncbi_42241	RAPGEF	0,083929503	0,978396516	0,99993434	1,198214046	0,008274015	0,176697752	1,114284543	0,000403691	0,017285321
ncbi_42242	PALLD	1,542010356	0,003212646	0,16714721	1,303342394	0,000960682	0,044953773	-0,238667961	0,711830875	0,94081146
ncbi_42243	CLCN3	0,646071714	0,279616858	0,99993434	1,097036634	0,000599979	0,032332477	0,45096492	0,232857435	0,703309736
ncbi_42245	GAB1	-0,003374727	0,797046292	0,99993434	1,223451421	0,002740846	0,090098469	1,226826148	0,000748266	0,027035977
ncbi_42248	SH2D4A	-1,358646317	0,004535112	0,21076168	0,545248914	0,37901554	0,847825521	1,903895232	0,000927646	0,031288479
ncbi_42254	CENPU	0,360021266	0,556092235	0,99993434	-0,88861132	0,026574788	0,315713321	-1,248632586	0,001728313	0,047584521
ncbi_42267	MAD2L1	0,304954396	0,629742836	0,99993434	-1,4253807	0,000292254	0,01986671	-1,730335096	5.88783362150562e-0	0,000100843
ncbi_42275	RASL11E	-0,250632939	0,233973575	0,99993434	-1,200872928	1.3504023550405e-05	0,001740828	-0,950239988	0,006128772	0,108331188
ncbi_42282	CD38	-2,201018771	0,086758255	0,98896947	-4,015463117	0,000560957	0,030354029	-1,814444347	0,540488976	0,897727515
ncbi_42290	SPON2	-1,240032097	0,007063382	0,28036689	-2,284954802	1.03080086792866e-06	0,0002259	-1,044922706	0,004567961	0,090183047
ncbi_42291	RNF103	-1,512878104	9.32574505388	0,01281946	0,283505701	0,7088904	0,948481261	1,796383804	1.12410377355898e-0	0,001158178
ncbi_42297	E2F8	0,428056446	0,25631852	0,99993434	-0,617681233	0,035877985	0,360566518	-1,045737679	0,000356746	0,016002606
ncbi_42297	CSRP3	3,929790998	1.51770791227	0,00038115	4,047638095	1.8741704063891e-08	8.8012380977179	0,117847097	0,807488299	0,963657597
ncbi_42300	KNL1	0,215574904	0,796126474	0,99993434	-1,097322951	0,000106285	0,009194371	-1,312897854	0,001260206	0,038205966
ncbi_42301	PLCB2	1,00503559	0,042500703	0,77192862	1,647483585	2.10060126773838e-05	0,002444319	0,642447995	0,015821299	0,196655314
ncbi_42304	WEE1	0,280834985	0,420952751	0,99993434	-0,884888605	0,001679905	0,0642124	-1,165723591	8.44167960261126e-0	0,005251362
ncbi_42304	SCUBE2	-0,070112844	0,587943537	0,99993434	-1,202256145	2.29095968286786e-05	0,002619463	-1,132143301	0,00031816	0,014655216
ncbi_42305	NRIP3	0,588186402	0,107178515	0,99993434	-1,452876255	8.78895603004049e-05	0,007970068	-2,041062657	1.40009752525792e-0	4.1032191473558
ncbi_42306	INSC	0,815071763	0,137597951	0,99993434	-1,306013606	0,025077276	0,310430854	-2,121085368	5.92589203838461e-0	1.4209211673130
ncbi_42319	CHRM4	8,196397213	0,00438569	0,206017	9,329422631	8.94371837245745e-07	0,00021778	1,133025419	0,049357548	0,360624565
ncbi_42320	ZFYVE19	-0,815264988	0,002370077	0,13755928	0,419838486	0,337246824	0,825783703	1,235103473	0,000478702	0,019545274
ncbi_42323	ZNF106	0,435277522	0,440836199	0,99993434	-1,601964996	0,000522236	0,029345669	-2,037242518	1.24066787399111e-0	3.718619982317e

ncbi_42325	DCAF4	-0,039720429	0,904367301	0,99993434	-2,324234562	7.22765035856971e-06	0,001059696	-2,284514133	0,060601129	0,393576124
ncbi_42327	PLEKHH	0,42647844	0,445569528	0,99993434	-2,539733247	6.36604830056381e-05	0,006154939	-2,966211687	2.16971590012108e-0	4.470970826687e
ncbi_42342	ASB2	1,633217669	0,032096425	0,67823173	2,618909833	1.17494762390324e-06	0,000248691	0,985692163	0,011772534	0,163945284
ncbi_42354	C5H14orf	-0,130703692	0,929961605	0,99993434	1,799217873	0,000482925	0,027711753	1,929921564	6.42634508678585e-0	0,004258826
ncbi_42355	WDHD1	0,639324072	0,04652015	0,79011686	-0,699741584	0,027757147	0,320437862	-1,339065655	2.45224503139486e-0	0,002156014
ncbi_42358	GNPNAT	0,167235914	0,931607586	0,99993434	-1,084766785	0,000380403	0,023932639	-1,252002699	0,000297052	0,014056698
ncbi_42361	LDB3	2,571941699	4.10957936070	0,0008656	2,950050813	0,000194233	0,014511225	0,378109114	0,634092968	0,919755616
ncbi_42363	ANXA11	-0,953346125	0,000990359	0,07220919	0,565862128	0,250560142	0,770005961	1,519208254	0,000359133	0,016055055
ncbi_42366	ARID5B	-0,768405015	0,000741189	0,05795922	0,701262507	0,076254571	0,503601883	1,469667522	4.91653535176271e-0	0,003430649
ncbi_42366	TMEM26	-1,910879758	6.005621146176	1.960685163	0,851669446	0,09001097	0,546291864	2,762549204	1.09783085793108e-1	6.5809969792704
ncbi_42366	RSFR	2,132643072	7.58220888260	0,00145612	2,631305726	7.8909673614307e-12	9.432575439586	0,498662654	0,125355176	0,548058591
ncbi_42368	MYPN	1,965784285	0,016216866	0,45838973	2,499845887	0,000238919	0,017073597	0,534061602	0,308762187	0,766702265
ncbi_42368	DNA2	0,66630039	0,184438741	0,99993434	-1,178918095	0,006868848	0,159120889	-1,845218485	8.31089465707959e-0	0,000127447
ncbi_42372	RPS24	0,09467918	0,966178605	0,99993434	1,635179309	0,000312717	0,020979152	1,540500129	0,003657317	0,077741573
ncbi_42375	HELLS	0,850355628	0,018895015	0,496976	-0,263439146	0,357306626	0,835249066	-1,113794774	0,001197883	0,036738794
ncbi_42375	PITX3	3,336283388	0,001080098	0,07665763	4,004501392	7.25322233631571e-06	0,001059696	0,668218004	0,239975061	0,710149214
ncbi_42378	C6H10O	1,861394762	0,000511181	0,04734409	2,171148143	9.07023922796866e-05	0,008058417	0,309753381	0,489698708	0,874258367
ncbi_42378	ACTA2	1,155780027	0,000548649	0,04948319	0,138986957	0,983704078	0,99770005	-1,01679307	0,000305495	0,014236274
ncbi_42380	KIF11	0,130768357	0,961301633	0,99993434	-0,97341829	0,000526024	0,029423807	-1,104186647	0,000606233	0,023377181
ncbi_42384	ANKRD2	3,058893689	0,001587578	0,10344872	3,615048227	1.03068963174208e-06	0,0002259	0,556154538	0,417286971	0,835104955
ncbi_42387	NT5C2	-0,470168701	0,018016036	0,48482606	0,558762301	0,047102761	0,410712337	1,028931002	1.29279840911252e-0	0,000187356
ncbi_42388	SORCS1	1,08917051	0,011421536	0,37760466	-0,487849489	0,202662846	0,725614786	-1,577019999	0,000365841	0,016190292
ncbi_42389	HABP2	-3,010569242	0,017618323	0,4793285	2,189680297	0,015346367	0,241172725	5,200249538	1.10511785712108e-0	2.4290490499521
ncbi_42389	NRAP	1,813268961	0,000417722	0,04019286	2,572618511	1.2891392295416e-07	4.095099747775	0,759349549	0,13964878	0,572454883
ncbi_42391	SHTN1	-1,187627003	0,022420951	0,55814026	0,639224095	0,330869847	0,824134801	1,826851098	0,001157512	0,035750038
ncbi_42392	EIF3A	0,557169881	0,303716073	0,99993434	1,650300992	0,00037857	0,023931784	1,093131111	2.67592228606771e-0	0,000352901
ncbi_42396	MKI67	0,011256455	0,733923293	0,99993434	-1,228053756	0,000708122	0,036802744	-1,239310212	0,014873537	0,18878942
ncbi_42396	TCERG1	-1,787193627	1.70839641823	0,00301486	-0,962280334	0,015801824	0,243188317	0,824913293	0,050053826	0,363485213
ncbi_42397	ALDH18	-0,110800674	0,445698498	0,99993434	-1,739790959	8.11874704988511e-13	1.186148943988	-1,628990285	6.86941481579574e-0	2.2096059168466
ncbi_42398	SLC40A1	0,486204544	0,090803233	0,99875623	-0,706341459	0,00532052	0,138533701	-1,192546003	7.37541298014345e-0	0,000817369
ncbi_42402	IQCA1	-5,977279923	0,000725282	0,05740274	0,91753784	0,514346571	0,903518185	6,894817763	0,000416401	0,017657543
ncbi_42410	GPR1	-1,088672347	0,000995302	0,07220919	0,60975599	0,183574601	0,702713952	1,698428337	5.67935678690843e-0	0,003840993
ncbi_42414	ATP5G3	-0,081637846	0,615156382	0,99993434	-1,446879864	0,001170201	0,051634143	-1,365242018	0,000651745	0,024390987
ncbi_42427	CCDC93	0,266417045	0,449013882	0,99993434	1,258511847	0,000341408	0,022125169	0,992094802	0,006144353	0,108331188
ncbi_42429	MCM6	0,583989679	0,04498993	0,78862214	-0,662114331	0,003870243	0,112092139	-1,24610401	6.75394815443714e-0	1.5626503203634
ncbi_42429	THSD7B	0,944823293	0,034820027	0,70056151	-0,95995964	0,128972702	0,625779356	-1,904782933	0,001035961	0,033322579
ncbi_42434	KIF14	-0,05684586	0,72343535	0,99993434	-1,05684586	0,000344582	0,022125169	-1	0,003410407	0,074218561
ncbi_42436	RGS1	2,634408569	0,091754531	0,99875623	-0,18662129	0,851506377	0,980045915	-2,821029859	2.67410072574539e-0	1.0686678900342
ncbi_42438	TNFSF4	-0,835613182	0,050584861	0,80559476	0,349521464	0,521737506	0,906368633	1,185134646	0,001724021	0,047565681
ncbi_42459	AKR1A1	0,683001137	0,009328136	0,33319747	-0,554464169	0,007017761	0,161041083	-1,237465305	1.50600904266891e-0	3.2559421729045
ncbi_42462	STIL	0,293933852	0,570606319	0,99993434	-0,750722028	0,027825987	0,320950794	-1,04465588	0,000864849	0,030014807
ncbi_42464	ORC1	0,818393194	0,139681226	0,99993434	-0,636281278	0,162355565	0,677503436	-1,454674472	0,001128911	0,035279794
ncbi_42465	GLIS1	-1,075013354	0,035422759	0,704088	1,054732733	0,059692594	0,455277211	2,129746087	2.03070945242461e-0	0,001834315
ncbi_42467	JUN	1,305439972	0,002053676	0,1259106	0,214921172	0,677793617	0,942458692	-1,0905188	0,001034997	0,033322579
ncbi_42468	ITGB3BP	-0,216423464	0,398362041	0,99993434	-1,418390934	0,000704679	0,03676912	-1,201967471	0,003788848	0,079565807

ncbi_42475	IWS1	-0,563581124	0,038553691	0,73714882	0,511680682	0,124543682	0,618438398	1,075261806	0,000914833	0,030995247
ncbi_42476	PARL	9,14635653	0,000413357	0,04019286	7,163230349	0,149950562	1	-1,983126181	0,151125169	0,590880146
ncbi_42483	ITM2C	-7,321928095	0,030196042	0,64872853	2,898853277	0,220148278	0,740853071	10,22078137	2.45448274414128e-1	1.2449891703744
ncbi_42485	GAL3ST2	-4,044394119	0,157889471	1	3,975196609	8.90104596963158e-05	0,007983243	8,019590728	2.23871591432836e-(	4.5421823812557
ncbi_42491	BCL6	0,558529093	0,239479316	0,99993434	1,423264703	0,000402774	0,024864201	0,86473561	0,016969571	0,204006106
ncbi_42494	CHRND	4,797012978	0,00610908	0,26156876	5,292781749	0,000446862	0,026313644	0,495768771	0,4348353	0,846630399
ncbi_42494	CAPN10	0,551710695	0,435667845	0,99993434	-1,125862956	0,000956751	0,044929729	-1,677573651	6.39168144855578e-(	2.1073373735888
ncbi_42495	KNG1	-7,022367813	0,025971965	0,60029714	1,791413378	0,207340247	0,730524358	8,813781191	3.16753456812987e-(	0,00261084
ncbi_42497	TTC14	-0,103038522	0,632292283	0,99993434	0,947862377	0,007935414	0,171899115	1,050900898	0,000771214	0,027713258
ncbi_42502	SSR3	0,12124374	0,994096618	0,99993434	-0,928130168	5.56389234749059e-05	0,005584704	-1,049373907	0,000136563	0,007700122
ncbi_42502	SLC33A1	0,337898637	0,411626407	0,99993434	-0,71774269	0,006431667	0,15387004	-1,055641327	1.84085971316817e-(	0,001685921
ncbi_42505	TCP11L2	-0,04479961	0,579362537	0,99993434	1,220537819	3.05224263658699e-06	0,000528078	1,265337429	2.05355983133316e-(	0,00027635
ncbi_42510	FAM83D	1,438252213	0,436578631	0,99993434	-8,770388596	0,029176	0,327552148	-10,20864081	2.10162639116212e-1	3.9594641209494
ncbi_42520	GTSE1	0,578592976	0,171277131	0,99993434	-0,87089858	0,005235264	0,136693124	-1,449491556	2.77638386174929e-(	0,002322741
ncbi_42530	NCAPH	0,894105451	0,008241636	0,30838834	-0,542704813	0,141422276	0,647922723	-1,436810264	6.37071439587137e-(	0,000105021
ncbi_42548	CDC34	-0,84347712	0,016721462	0,4668494	0,796347316	0,043623372	0,396135165	1,639824436	3.51970407508314e-(	0,000429795
ncbi_42550	KCTD12	-0,573691388	0,166540409	0,99993434	1,237667686	0,001021103	0,046945746	1,811359074	5.18418417339265e-(	0,000615937
ncbi_42550	CMKLR1	0,356097235	0,423270786	0,99993434	1,172572149	0,000216932	0,015759316	0,816474914	0,00282751	0,066115611
ncbi_42563	CDCA7	0,32457537	0,501911654	0,99993434	-1,060521619	0,002927749	0,09321299	-1,38509699	0,00017798	0,009573702
ncbi_42585	VPS33B	0,791937316	0,048313077	0,79148427	1,122903436	0,00034359	0,022125169	0,33096612	0,36664293	0,804960507
ncbi_42604	SAFB	0,099946288	0,976889264	0,99993434	1,729802125	0,003957661	0,11412123	1,629855837	3.62882059900965e-(	0,002865682
ncbi_42607	FRMD4B	0,204280372	0,627184333	0,99993434	-1,351207235	8.92875591935462e-07	0,00021778	-1,555487607	2.31427464398853e-1	1.2208261601968
ncbi_42620	LRRC2	6,129283017	0,007056524	0,28036689	6,857980995	1.59688826179736e-05	0,00199976	0,728697978	0,460794059	0,858857159
ncbi_42629	MXD1	-0,356290657	0,353064206	0,99993434	0,930436406	0,021997696	0,290993673	1,286727063	3.87821387071675e-(	0,00295641
ncbi_42635	S100A9	0,954088328	0,333139292	0,99993434	4,59483116	2.69572358424094e-17	1.77230347045921	3,640742832	2.83658232470861e-(	0,000366753
ncbi_42656	CENPL	0,327384817	0,672789	0,99993434	-1,516744272	0,006898863	0,159338923	-1,844129089	0,000279308	0,013592303
ncbi_42675	TRIM63	3,077691285	6.22707741177	0,00924084	3,524589883	2.67147977868005e-08	1.13313830999561	0,446898598	0,299351558	0,759103159
ncbi_42676	MCM4	0,544047196	0,117979578	0,99993434	-0,561245302	0,026703332	0,316326223	-1,105292498	4.32350521058094e-(	0,003167688
ncbi_42681	DMBT1	1,553340522	0,021669065	0,5500101	2,544320516	0,000422144	0,02581755	0,990979995	0,097122996	0,489624644
ncbi_42682	LOC4268	0,242458044	0,767020125	0,99993434	1,808701348	0,000991156	0,046162998	1,566243303	0,000531842	0,02138395
ncbi_42684	LIPG	1,06221898	0,003839249	0,18991195	-1,486619447	1.0707460574002e-10	8.79952494297198	-2,548838428	1.86122578437765e-1	2.0454871370310
ncbi_42688	RACGAF	0,197307292	0,797668239	0,99993434	-1,101835322	6.89175967930222e-05	0,006530214	-1,299142614	3.21056963885377e-(	0,002613642
ncbi_42689	AQP3	-0,074957948	0,572007697	0,99993434	-2,044124468	1.88519192535398e-14	4.13139810441324	-1,969166519	1.58866591531565e-1	1.9046660082893
ncbi_42693	FIBIN	0,183712377	0,647330535	0,99993434	-1,366085486	8.74783919918306e-08	3.02698256921205	-1,549797863	1.90659902126399e-(	3.9911472845126
ncbi_42699	ROGDI	-0,770581205	0,034222086	0,69604961	0,609055217	0,067451976	0,480458305	1,379636422	0,000273931	0,013429744
ncbi_42704	SAFB2	-0,346900623	0,547447776	0,99993434	1,301528296	0,317273523	0,817410739	1,64842892	0,001293497	0,038857954
ncbi_42710	CETN3	-0,253626953	0,53470016	0,99993434	1,267661803	0,027750445	0,320437862	1,521288756	0,000809003	0,028375358
ncbi_42717	CMYA5	1,949118831	1.91440098577	8.33338749101	3,088302838	8.39200471869652e-07	0,000212205	1,139184007	0,064867259	0,406784856
ncbi_42728	CPLX1	1,478047297	0,005687576	0,25008097	-0,509674373	0,371575615	0,841806989	-1,98772167	8.80820957030454e-(	0,005453646
ncbi_42729	HOOK3	-1,169925001	0,003264418	0,16849816	1,30580843	0,029035126	0,327552148	2,475733431	0,000424138	0,017927981
ncbi_42731	RNF20	0,155232718	0,836576005	0,99993434	1,232945387	0,000193677	0,014511225	1,077712669	0,000322522	0,014748305
ncbi_42746	CTSV	-0,12753382	0,626357961	0,99993434	1,14887096	0,009909335	0,193100357	1,27640478	3.31113746665641e-(	0,002662639
ncbi_42749	C2CD4A	3,183599938	0,005449117	0,24204086	3,786596362	1.71889783794884e-05	0,002073559	0,602996424	0,504466891	0,882699366
ncbi_42750	AGBL1	1,852116373	0,000109817	0,0147845	1,408392186	0,008216201	0,176239515	-0,443724187	0,381555437	0,813904701
ncbi_42754	CEBPA	2,584962501	0,04359459	0,77879856	3,696967526	0,000201234	0,014865283	1,112005026	0,144219323	0,578739615

ncbi_4276(	PSD2	0,241906011	0,57227706	0,99993434	-1,471482646	0,000124331	0,010347015	-1,713388658	2.9991295063826e-06	0,000376691
ncbi_4276	PDE8B	-1,7589919	0,168093172	0,99993434	1,334984248	0,080821077	0,51974338	3,093976148	0,00114556	0,035547399
ncbi_4276(	C1QTNF	0,00641355	0,90911669	0,99993434	-1,261554267	0,000156862	0,012425137	-1,267967817	1.01689394542501e-(	0,001090309
ncbi_4277	MYH1G	2,781359714	4.82526459141	0,0009664	3,078341451	1.30804007458021e-07	4.0950997477750	0,296981738	0,420381151	0,836436008
ncbi_4278(	RAB11FI	0,070458095	0,963975168	0,99993434	-1,051451532	0,00055725	0,03028992	-1,121909626	0,000153088	0,008342655
ncbi_4278	GSG2	0,512385614	0,264346649	0,99993434	-0,748461233	0,05528795	0,44139724	-1,260846847	0,001484871	0,042386326
ncbi_4279	ZFP2	-4,614709844	0,000102404	0,01393018	0,914069821	0,283527519	0,797967327	5,528779665	6.12678213404e-06	0,000702609
ncbi_4279	MCAT	4,656623486	2.17168348172	0,0036359	4,718818247	0,00011592	0,009897634	0,062194762	0,802286878	0,963292147
ncbi_4280	GJB2	0,369723606	0,477353345	0,99993434	-1,514573173	0,036374463	0,361791652	-1,884296779	0,000744744	0,026982653
ncbi_4281	HBE1	-1,209330358	0,261614734	0,99993434	1,565809939	0,05701206	0,447368304	2,775140297	0,000959485	0,032034659
ncbi_4281	MATN4	-2,780964162	6.32174548615	6.87963952	-0,397888622	0,246042647	0,766875521	2,383075541	6.48977491746366e-1	1.7117430322302
ncbi_4281	HRH3	-3,406505866	1.06755793053	7.33749421	0,816858395	0,04078779	0,385563373	4,22336426	5.88730751713669e-1	9.7052264419998
ncbi_4281(	AURKA	0,442019831	0,534479002	0,99993434	-1,282310507	0,010960265	0,201280065	-1,724330338	0,001286783	0,038833167
ncbi_4281	PADI2	2,158429363	0,08293334	0,97899918	-2,807354922	0,301642638	1	-4,965784285	0,000727842	0,026589429
ncbi_4282(	HIVEP3	-0,735966844	0,001600172	0,10344872	1,178778635	0,002349804	0,081095981	1,914745479	2.38280598270353e-(	6.2848890599788
ncbi_4282	HEPACA	1,652303197	8.50269331215	0,01193943	-0,751154783	0,21569874	0,73759978	-2,40345798	7.25956347144667e-(	0,00469253
ncbi_4283(	MCMDC	-3,162938571	7.33082537846	0,01052014	-1,178705887	0,017876156	0,26001501	1,984232684	0,040500223	0,330722566
ncbi_4284(	ALS2CL	-0,869991013	0,005064202	0,23043	0,448654498	0,313108832	0,815752342	1,318645511	0,000932052	0,0313569
ncbi_4285	CRLS1	-0,021660172	0,65570471	0,99993434	-1,104810214	0,00089672	0,042720926	-1,083150042	0,002266982	0,056838312
ncbi_4286	CLVS2	1,165586066	0,067321614	0,90561458	-0,509013647	0,461415075	0,887428688	-1,674599713	7.40540967918417e-(	0,004735114
ncbi_4286	CNR1	0,692332598	0,199620517	0,99993434	-1,307667402	0,045996951	0,405057231	-2	0,000537521	0,021546584
ncbi_4286	LUC7L3	-0,408280133	0,405021764	0,99993434	1,995760517	0,000788309	0,039114988	2,40404065	7.29427263933669e-(	0,00469253
ncbi_4287	SPRY3	1,39218656	2.84775683395	0,0006361	0,268748057	0,4287561	0,876689456	-1,123438503	0,001473517	0,042356051
ncbi_4287	CDKL2	-1,490607066	0,000566416	0,04997854	-0,142757901	0,734367695	0,956824623	1,347849165	0,01665999	0,202499489
ncbi_4287	NWD2	0,037089319	0,893703542	0,99993434	-1,10922907	0,000500722	0,02825747	-1,146318388	0,003068534	0,069294226
ncbi_4289	PRDX3	-0,721926195	0,108949392	0,99993434	0,64143527	0,190343897	0,709808851	1,363361465	4.78287947492835e-(	0,003376069
ncbi_4289	LRRFIP1	0,703606997	0,412514713	0,99993434	2,826851098	1.78363420179652e-05	0,002132091	2,1232441	4.44928914955929e-(	0,003229845
ncbi_4289	ACKR3	-0,797287719	0,002829978	0,15793455	1,201325593	0,00696416	0,160090449	1,998613312	6.6575984994618e-06	0,000744071
ncbi_4291	VPS8	-1,386823897	0,000337267	0,03387981	-1,559291093	2.8102830229266e-06	0,000499357	-0,172467196	0,705038031	0,93902011
ncbi_4292	MYO7L2	3,052568051	0,001791377	0,11411509	2,817783122	5.23165223080986e-07	0,000137582	-0,234784929	0,682516133	0,931800497
ncbi_4292	USP13	1,49683159	0,000443596	0,04228411	2,242179043	9.60774216191722e-07	0,0002259	0,745347453	0,0423102	0,336342952
ncbi_4296	PRKAG1	0,757649748	0,100085094	0,99993434	1,832189314	0,00014981	0,012097934	1,074539566	0,050079817	0,363485213
ncbi_4304	VAT1L	-1,251280736	0,003203695	0,16714721	0,509989739	0,333107923	0,825221021	1,761270474	0,000667902	0,024812088
ncbi_4304	SLC39A	-1,095064307	0,001392423	0,09470651	-1,059120867	8.9249119766154e-05	0,007983243	0,03594344	0,765346662	0,954403729
ncbi_4305	ACACB	1,751740947	0,000921692	0,06800214	1,837349662	3.51090241407117e-05	0,00375324	0,085608716	0,870287402	0,976297232
ncbi_4306	EDRF1	0	1	1	9,840253403	2.87056593511986e-09	1.7156850673132	9,840253403	9.20024054183543e-1	4.3333132952044
ncbi_4309	ABRA	4,359895945	4.18975545887	1.478757203	3,180572246	0,009735648	0,191185352	-1,179323699	0,15923082	0,603247281
ncbi_4310(	LOC4310	-1,013235827	0,18582102	0,99993434	-7,77478706	9.52555717868349e-09	5.0100620537003	-6,761551232	3.49525240835556e-(	0,000429795
ncbi_4314	MCHR2	-2,48624445	0,000130457	0,01670237	0,440357472	0,437834282	0,879307274	2,926601922	1.65854723904817e-(	0,001573591
ncbi_4316(	NPR3	1,952034675	4.75386239514	0,0009664	1,352048083	0,002275027	0,079771564	-0,599986591	0,105409495	0,50689785
ncbi_6932	CENPN	0,549801661	0,381295902	0,99993434	-1	0,065498446	0,473468428	-1,549801661	0,001849855	0,049991573
ncbi_7687	TIMP4	-0,534313492	0,037951752	0,73286538	1,420266014	0,003096263	0,09771816	1,954579506	3.94278186942894e-(	0,00297128
ncbi_7687	C9H2OR	-0,231041371	0,287433309	0,99993434	0,862806319	0,025769054	0,311670061	1,093847689	4.81271627820935e-(	0,003376069
ncbi_7689	CD80	2,252387162	8.12954742187(	0,01153954	1,565979397	0,00319145	0,098739706	-0,686407764	0,179076159	0,629804439
ncbi_7690(	MEF2C	-1,220298524	0,000604996	0,05267096	0,45615289	0,380936531	0,848427096	1,676451414	0,000382039	0,016573451

ncbi_76902	PABPN1I	1,432959407	0,290141076	0,99993434	-7,058893689	0,043746984	1	-8,491853096	0,000793028	0,028114128
ncbi_7690(	TP53INP:	-0,595670929	0,04171355	0,76593014	0,513953562	0,170165691	0,689833673	1,109624491	7.48981835907403e-(	0,00011759
ncbi_7692(	PYCR1	0,304114788	0,443816643	0,99993434	-1,384151409	1.21045101113875e-06	0,000248691	-1,688266196	4.27032444513641e-(	0,00051667
ncbi_7692:	IGHVL	-4,781359714	0,274665676	1	1,678071905	0,202117857	0,724747125	6,459431619	0,001106118	0,034981966
ncbi_76941	SPTB	0,623436649	0,217962442	0,99993434	1,834940754	4.79976742948233e-05	0,004930636	1,211504105	0,001376377	0,040253807
ncbi_76942	PRKAR2	0,906564526	0,007864645	0,29942972	1,593090382	0,000737695	0,037644612	0,686525856	0,616742447	0,916771797
ncbi_76942	HS3ST3E	-0,991444848	3.46907266695(	0,00559292	0,921978162	6.24911459475858e-05	0,006086638	1,91342301	7.62315740832303e-1	1.0053419990096
ncbi_7694(	HS3ST3E	-0,251893848	0,439234606	0,99993434	1,165361476	0,004465653	0,124404376	1,417255325	3.51792926911442e-(	0,002794846
ncbi_76951	LOC7695	0,803752685	0,245307082	0,99993434	-0,840103504	0,032393255	0,341297199	-1,64385619	0,000412239	0,017537445
ncbi_76967	ERNI	-3,865198997	1.46023433386:	1.362085726	-1,245679274	0,230247379	0,753237531	2,619519723	0,004354341	0,087362147
ncbi_7698:	PFKL	0,319281757	0,41006491	0,99993434	-0,816527552	0,006448101	0,15387004	-1,135809308	0,001820123	0,049390489
ncbi_76988	APOLD1	1,819710978	1.491531673204	0,00274337	-0,443002508	0,320393288	0,817874818	-2,262713486	2.22548449978579e-(	9.4676418010241
ncbi_76992	LOXL4	-3,302913717	9.426448609734	3.095102313	0,290028529	0,462601551	0,887616727	3,592942246	1.89812944630958e-2	1.2516265568965
ncbi_7699:	NDUFB4	-0,499871407	0,332737919	0,99993434	0,941673297	0,071572294	0,492051003	1,441544703	0,000986202	0,032433986
ncbi_7701:	ZNF503	-0,339024089	0,384759029	0,99993434	1,051927792	0,014765321	0,23763672	1,390951881	0,000215544	0,011147428
ncbi_77042	BICDL1	2,259867127	0,008693623	0,31890456	-0,788495895	0,576111353	0,920347355	-3,048363022	0,001114201	0,035153312
ncbi_77051	CDCA3	0,121008323	0,987399578	0,99993434	-1,962920058	1.0551501860077e-05	0,001430327	-2,083928382	4.10705966870805e-(	1.0219604322815
ncbi_77058	HRG	-2,415037499	0,3433788	1	2,285402219	0,047615579	0,412448783	4,700439718	0,000427934	0,017973231
ncbi_7707:	PRODH	1,405638801	0,043101411	0,77502048	2,260690465	0,000105043	0,00914706	0,855051664	0,090123369	0,471768417
ncbi_77077	IRX5	-4,282933963	3.007099794721	1.869986486	1,023279661	0,138605236	0,642665613	5,306213624	0,000323192	0,014748305
ncbi_7708(	SQSTM1	0,145574277	0,827394945	0,99993434	1,259129436	5.98184692987111e-06	0,000914597	1,113555159	3.74491131609154e-(	0,00290517
ncbi_7708:	ANGPTL	-1,086562666	8.392206539291	0,00158832	0,101365601	0,94193689	0,992037984	1,187928267	0,002178622	0,055466542
ncbi_7708(	GPC3	-1,894817763	0,090143863	0,99846371	1,918963428	0,051727355	0,430482907	3,813781191	0,000103428	0,006123189
ncbi_77092	DGUOK	0,718028535	0,098067836	0,99993434	-1,57884073	1.88967636243018e-05	0,0022385	-2,296869265	4.41810120805139e-1	4.1618513379844
ncbi_7710:	BMP8A	-1,383512598	4.958194187138	0,0009664	1,410694422	0,000652725	0,034747705	2,79420702	4.27160555373952e-1	2.9649438969851
ncbi_77111	PRR33	2,660911354	0,001510469	0,10063888	3,290392521	1.12148301341902e-05	0,001489533	0,629481167	0,212848442	0,675909952
ncbi_77144	SRM	0,743365364	0,00997694	0,34546727	-0,474044115	0,029113729	0,327552148	-1,217409479	2.77048794007134e-(	0,000361754
ncbi_77147	LMOD1	0,871379834	0,18485108	0,99993434	1,821830245	0,000234514	0,016850411	0,950450411	0,052203407	0,370117005
ncbi_77147	ATP5D	-0,318164094	0,253535417	0,99993434	0,785655896	0,039638128	0,379055815	1,10381999	0,000739588	0,026895764
ncbi_77151	HAUS3	0,347128649	0,542599719	0,99993434	-0,943905438	0,001457412	0,058964659	-1,291034087	0,001125358	0,035279794
ncbi_77167	GUCY1A	0,770518154	0,010851978	0,36713983	-0,249519599	0,366446489	0,838674818	-1,020037753	0,000284149	0,013676484
ncbi_77177	REEP1	8,395177077	0,047797865	0,79011686	7,64385619	1.10781817377004e-05	0,001486398	-0,751320887	0,612638052	0,9155244
ncbi_77181	LOC7718	-0,475730889	0,031499635	0,66778204	0,596628562	0,063354221	0,466691684	1,072359451	9.37298647347041e-(	0,005670227
ncbi_77184	TM4SF18	1,544079444	0,000208135	0,02405338	0,582147485	0,264978859	0,784793986	-0,961931959	0,024812279	0,25495997
ncbi_77198	SYTL1	-1,953349907	0,050282911	0,80559476	0,861480136	0,385166398	0,850899357	2,814830043	1.42929051030813e-(	0,001396258
ncbi_7721:	NDUFA1	-0,061575279	0,842827836	0,99993434	2,419050562	0,000717653	0,036861036	2,480625841	0,009143714	0,141534392
ncbi_7721:	LOC7721	3,384049807	0,002902937	0,16063327	3,276198865	2.70144204968529e-05	0,00298498	-0,107850942	0,998587825	0,999421435
ncbi_7722:	LHX6	-0,951947871	0,000563212	0,04997854	0,688322002	0,413907896	0,867968806	1,640269872	0,000233576	0,011757256
ncbi_77228	NDC1	0,532411979	0,206304793	0,99993434	-1,140498221	0,001740358	0,065758516	-1,6729102	2.55178746430547e-(	0,002199541
ncbi_7723(	ANXA4	-0,911544487	0,000120965	0,01579676	0,190411458	0,687715181	0,943199185	1,101955945	0,000704225	0,026014897
ncbi_7760(	FBXO39	-0,137927908	0,353645332	0,99993434	-1,191298652	8.65658818324523e-06	0,001237233	-1,053370744	0,000376293	0,016382026
ncbi_77614	HOXA10	-1,22485562	0,229898277	0,99993434	-1,428389014	1.6690060194142e-05	0,002051006	-0,203533394	0,011153573	0,16005802
ncbi_7765:	NRBP2	-0,681755608	0,039846073	0,74978368	0,858615167	0,015594432	0,242951637	1,540370774	1.85603060784164e-(	3.9479567187444
ncbi_7766:	SEC61G	0,192755642	0,811591239	0,99993434	-1,187725289	0,000331874	0,021928673	-1,380480931	8.13826747848149e-(	0,005096727
ncbi_7768:	AKAP8L	0,259476586	0,894105546	0,99993434	1,353698752	0,001105205	0,049768283	1,094222166	0,012993214	0,172910702

ncbi_77758	WNK4	-0,969684376	0,00775336	0,29699688	1,312427454	3.51482639152392e-06	0,000592519	2,28211183	5.96935621906218e-1	3.9361934908496
