# Supplementary figures and images for: BMP8A, TGF-β1 regulates chicken chondrocyte proliferation, differentiation, and apoptosis induced by Thiram
Source: Anim Biosci. 2025 Sep 30;39(1):250413. doi: 10.5713/ab.25.0413 (PMC12754447; doi:10.5713/ab.25.0413)

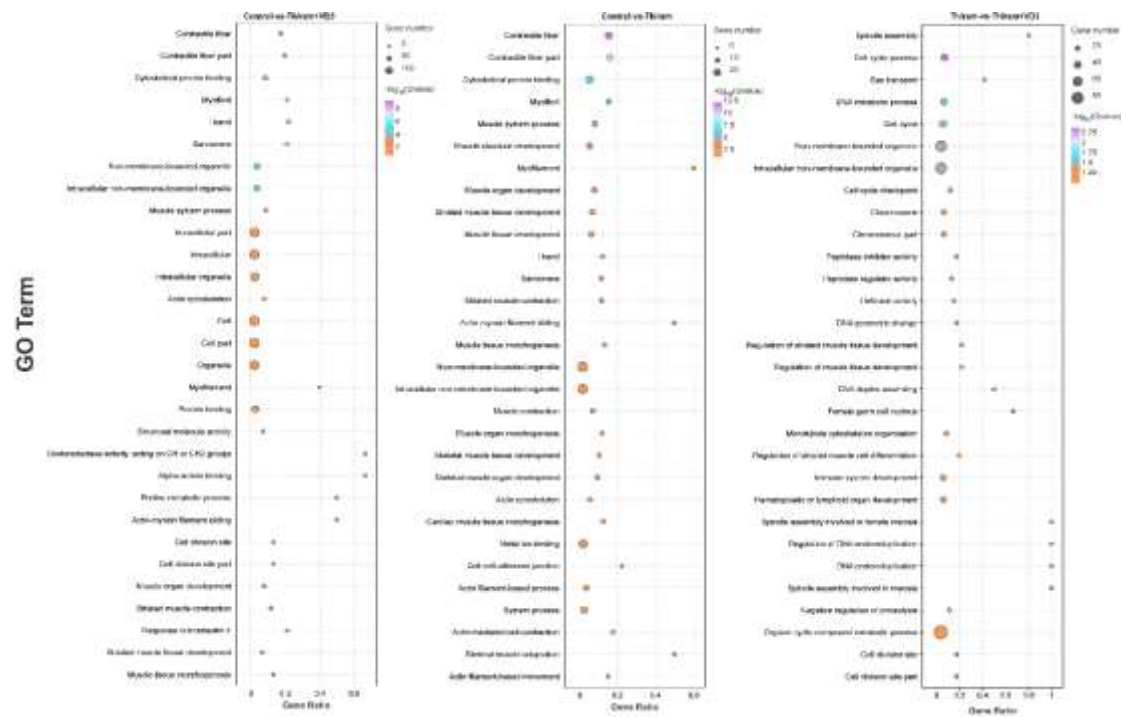

**Supplement 6. Delineation of gene ontology analysis of differentially expressed genes (DEGs).**

Supplement: Supplementary file 6 [file ab-25-0413-Supplementary-6.pdf]

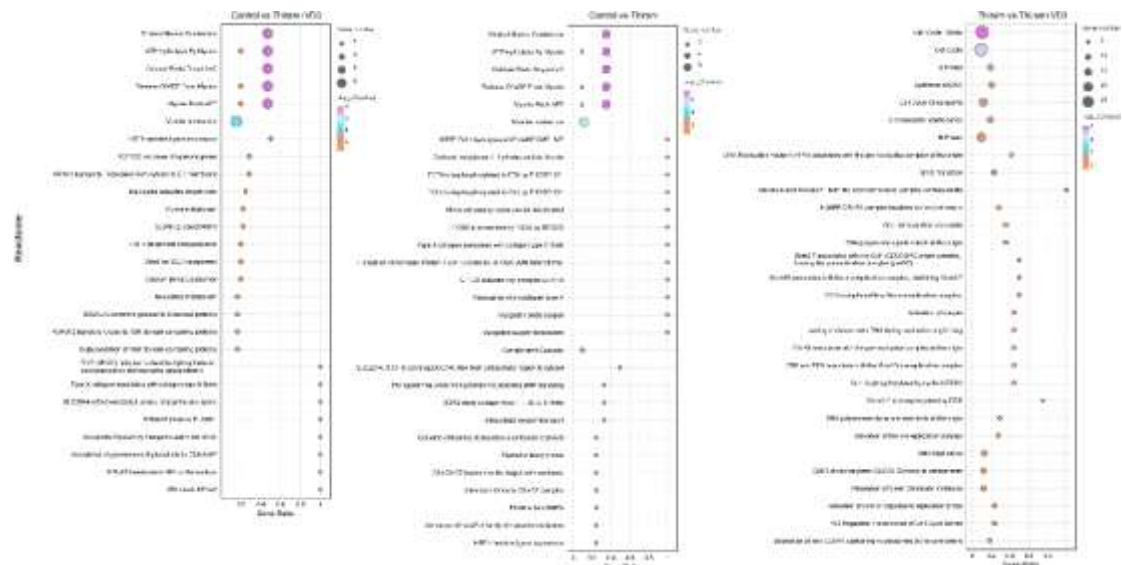

Supplement 8. Reactome analysis of differentially expressed genes (DEGs).

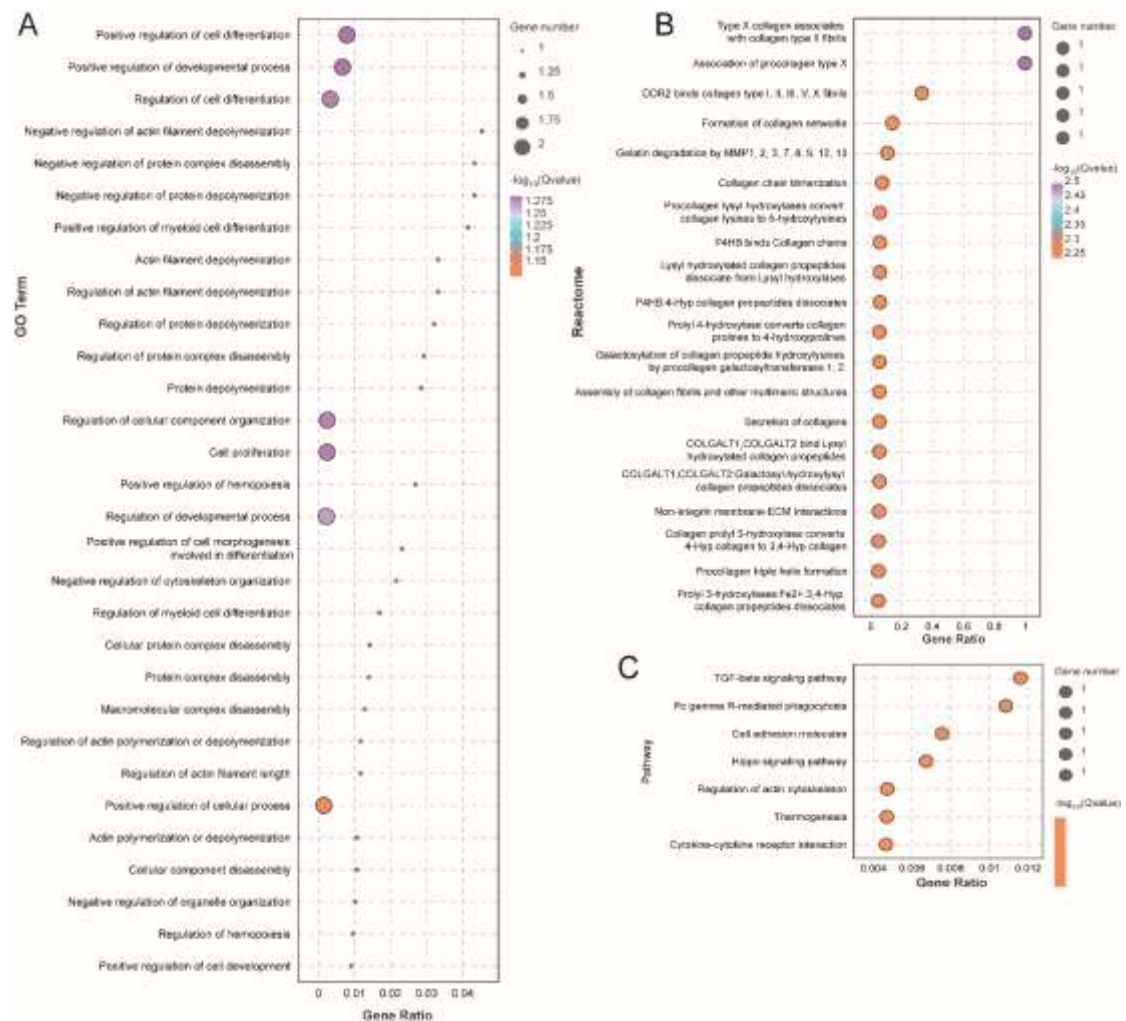

Supplement: Supplementary file 8 [file ab-25-0413-Supplementary-8,9.pdf]
