# Supplementary material for: BMP8A, TGF-β1 regulates chicken chondrocyte proliferation, differentiation, and apoptosis induced by Thiram
Source: Anim Biosci. 2025 Sep 30;39(1):250413. doi: 10.5713/ab.25.0413 (PMC12754447; doi:10.5713/ab.25.0413)
Supplement: Supplementary file 7 [file ab-25-0413-Supplementary-7.pdf]

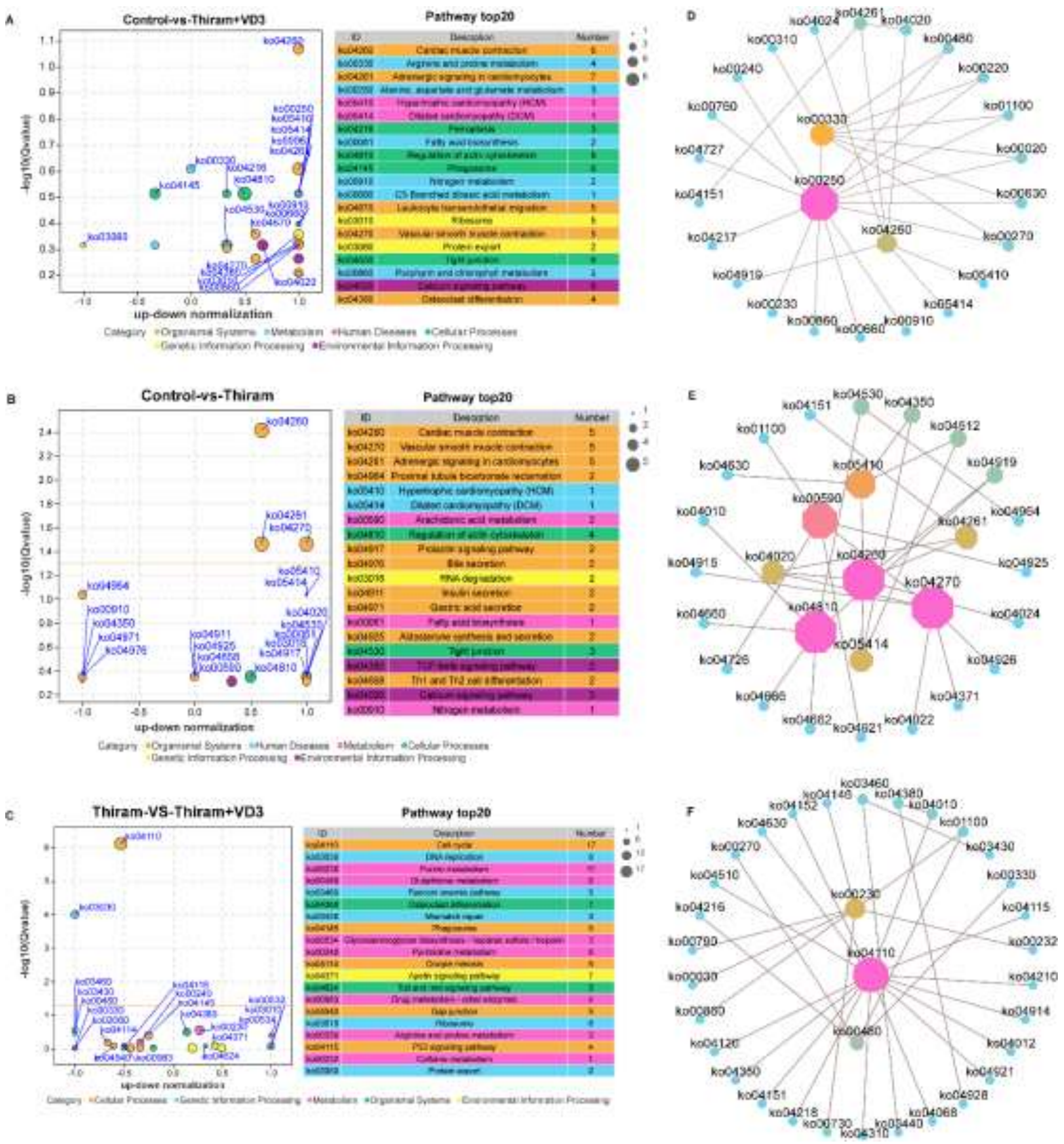

Supplement 7. KEGG pathway analysis of differentially expressed genes (DEGs). Top 20 DEMs KEGG comment and pathway network of the comparison groups on *Control-vs-Thiram+VD3* (A, D), *Control-vs-Thiram* (B, E), and *Thiram-vs- Thiram+VD$_3$* (C, F). The size of the circle represents the number of enriched DEGs.
