# Supplementary material for: BMP8A, TGF-β1 regulates chicken chondrocyte proliferation, differentiation, and apoptosis induced by Thiram
Source: Anim Biosci. 2025 Sep 30;39(1):250413. doi: 10.5713/ab.25.0413 (PMC12754447; doi:10.5713/ab.25.0413)
Supplement: Supplementary file 9 [file ab-25-0413-Supplementary-10.pdf]

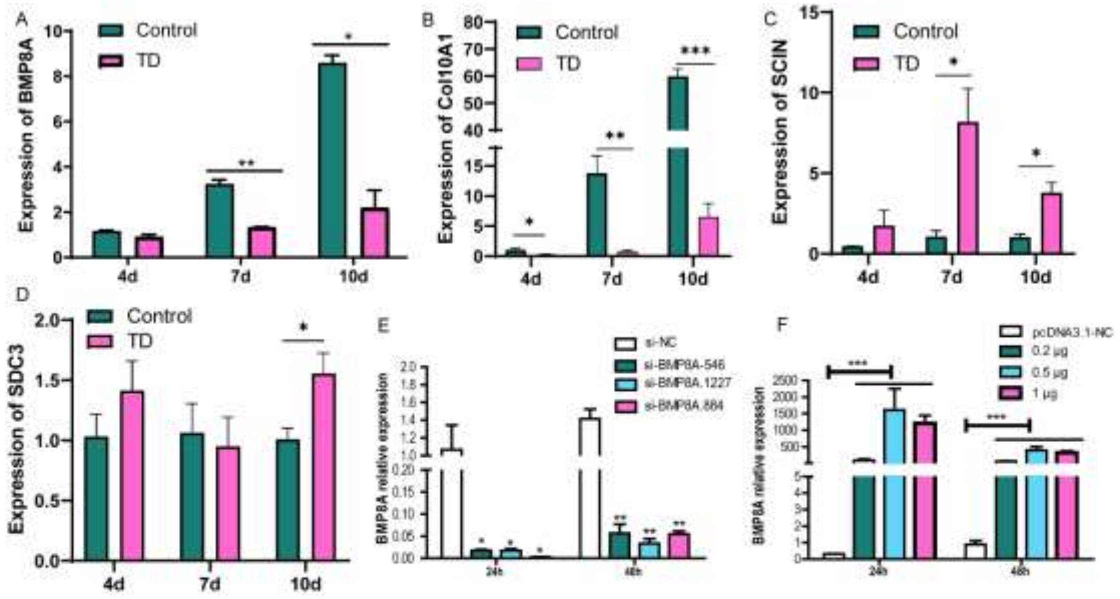

Supplement 10. Relative expression of co-DEGs. The expression levels of *BMP8A* (A), *COL10A1*(B), *SDC3*(C), and *SCIN* (D) at the growth point of chondrocytes and the screen of si-BMP8A (E) and pcDNA3.1-BMP8A (F). The data was presented as mean±SEM for n=3 (Values represent the mean of three technical replicates), * P<0.05, ** P<0.01, *** P<0.001.
