# Supplementary material for: BMP8A, TGF-β1 regulates chicken chondrocyte proliferation, differentiation, and apoptosis induced by Thiram
Source: Anim Biosci. 2025 Sep 30;39(1):250413. doi: 10.5713/ab.25.0413 (PMC12754447; doi:10.5713/ab.25.0413)
Supplement: Supplementary file 10 [file ab-25-0413-Supplementary-11.pdf]

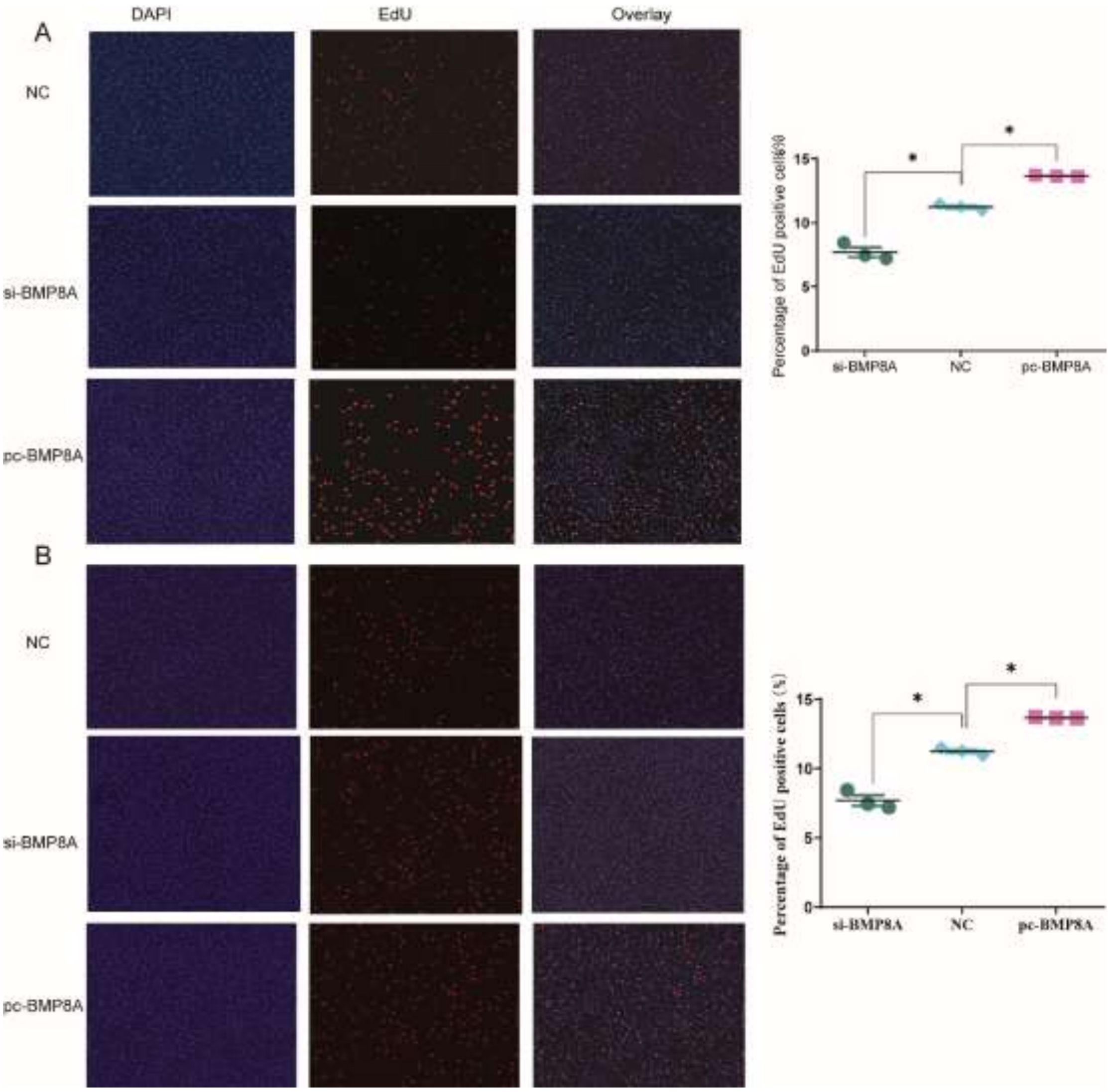

Supplement 11. Chondrocytes proliferation performed by EdU. EdU assays for TD (A) and control (B) chicken chondrocytes that were transfected with si-BMP8A, si-NC, pc-BMP8A and pc-NC for 48h (n=3). EdU fluorescence, which is depicted in red, marks proliferating cells. DAPI fluorescence, shown in blue, highlights the nuclei of the cells. All photomicrographs were taken at a magnification of 100×. On the right side of the figure, the quantified results of EdU labeling are displayed. * $P<0.05$.
