# Supplementary material for: BMP8A, TGF-β1 regulates chicken chondrocyte proliferation, differentiation, and apoptosis induced by Thiram
Source: Anim Biosci. 2025 Sep 30;39(1):250413. doi: 10.5713/ab.25.0413 (PMC12754447; doi:10.5713/ab.25.0413)
Supplement: Supplementary file 11 [file ab-25-0413-Supplementary-12.pdf]

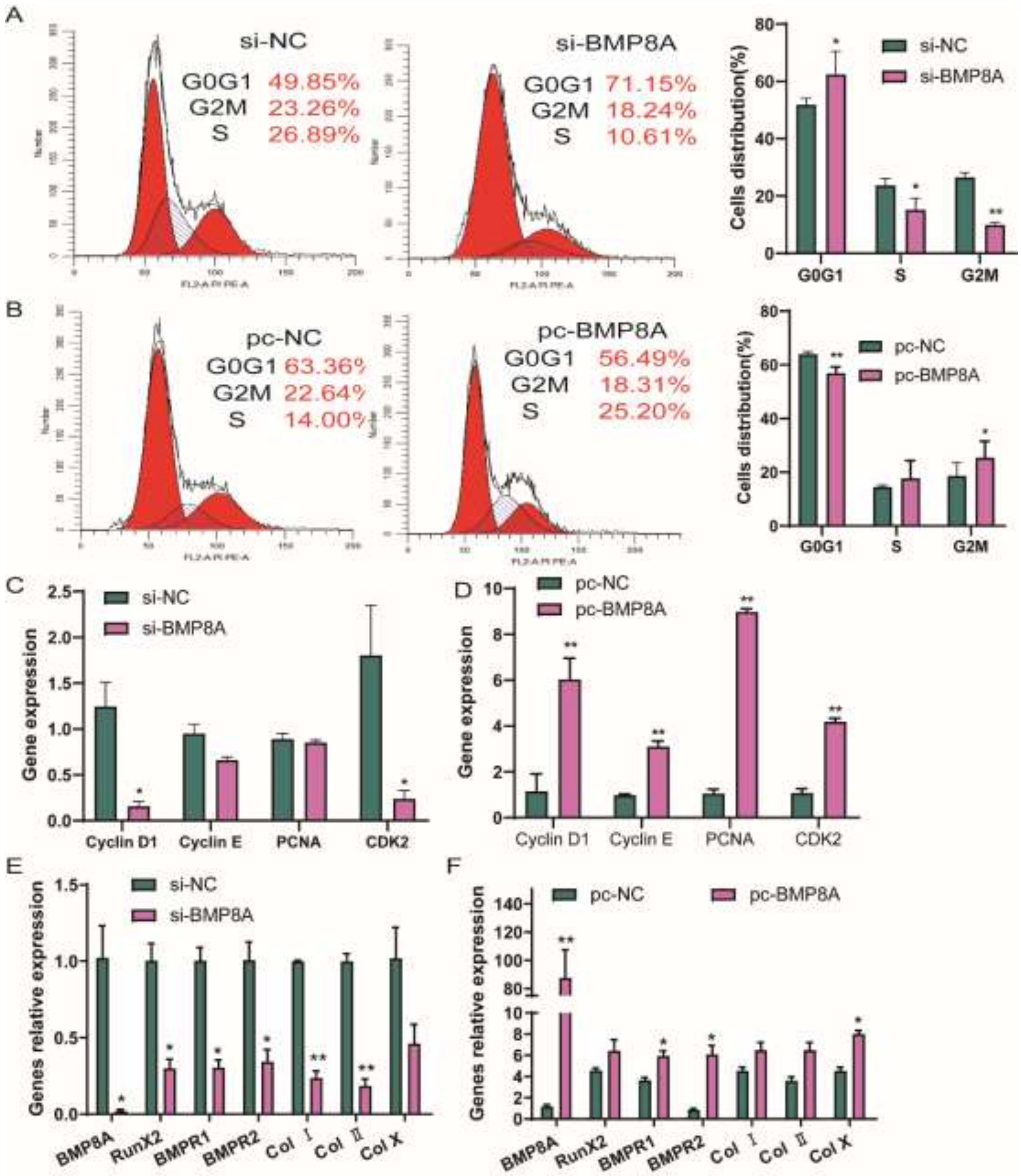

Supplement 12. BMP8A promoted chondrocytes proliferation on the control broiler chickens. Flow cytometry for cell cycle analysis of the control chicken chondrocytes at 48 h after being knocked BMP8A compared to si-NC (A), and overexpressed BMP8A compared to pc-NC (B). The expression level of *Cyclin D1*, *Cyclin E*, *PCNA*, and *CDK2* was determined by RT-qPCR in the control chicken chondrocytes after transfection with si-BMP8A and si-NC (C), and pc-BMP8A and pc-NC (D). The expression level of *BMP8A*, *Runx2*, *BMPR1*, *BMPR2*, *Col I*, *Col II*, and *Col X* were determined by RT-qPCR in the control chicken chondrocytes after transfection with si-BMP8A
