# Supplementary material for: BMP8A, TGF-β1 regulates chicken chondrocyte proliferation, differentiation, and apoptosis induced by Thiram
Source: Anim Biosci. 2025 Sep 30;39(1):250413. doi: 10.5713/ab.25.0413 (PMC12754447; doi:10.5713/ab.25.0413)
Supplement: Supplementary file 12 [file ab-25-0413-Supplementary-13.pdf]

and si-NC (E), and pc-BMP8A and pc-NC (F). NC represents negative control or pcDNA3.1-NC, si represents si-BMP8A, and pc represents pcDNA3.1-BMP8A. The data was presented as mean±SEM for n=3 (Values represent the mean of three technical replicates), * $P<0.05$, ** $P<0.01$.

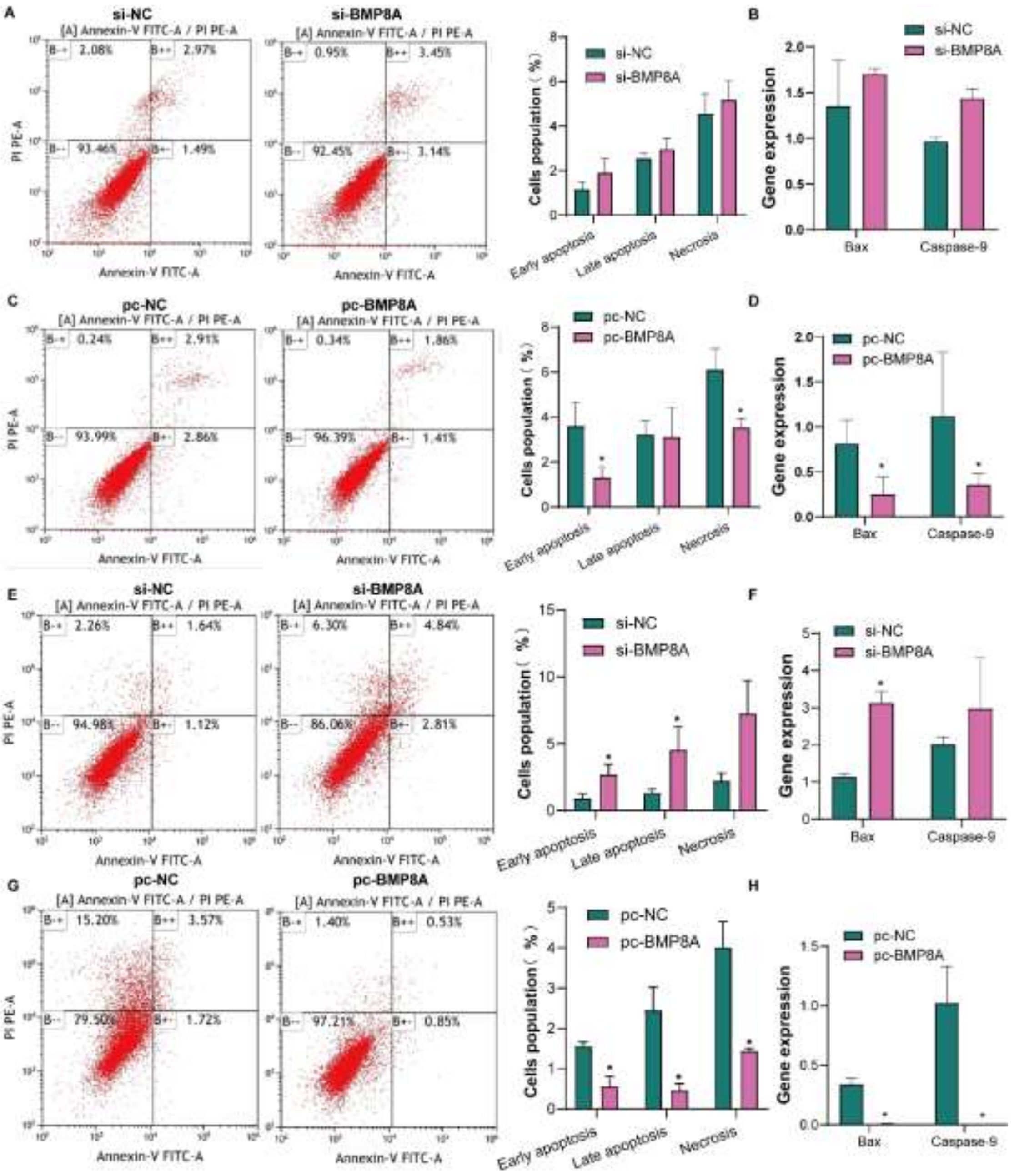

Supplement 13. Effect of BMP8A on chicken chondrocytes apoptosis. Apoptosis rate was performed by flow cytometry after being transfected with si-BMP8A and si-NC in control (A) and TD (E) chicken chondrocytes. The mRNA level of *Bax* and *Caspase-9*, was performed by RT-qPCR after being
