# Supplementary material for: BMP8A, TGF-β1 regulates chicken chondrocyte proliferation, differentiation, and apoptosis induced by Thiram
Source: Anim Biosci. 2025 Sep 30;39(1):250413. doi: 10.5713/ab.25.0413 (PMC12754447; doi:10.5713/ab.25.0413)
Supplement: Supplementary file 13 [file ab-25-0413-Supplementary-14.pdf]

transfected with si-BMP8A and si-NC in control (B) and TD (F) chicken chondrocytes. Apoptosis rate was performed by flow cytometry after being transfected with pc-BMP8A and pc-NC in control (C) and TD (G) chicken chondrocytes. The mRNA level of *Bax* and *Caspase-9*, was performed by RT-qPCR after being transfected with si-BMP8A and si-NC in control (D) and TD (H) chicken chondrocytes. The data was presented as mean±SEM for n=3 (Values represent the mean of three technical replicates), * $P<0.05$.

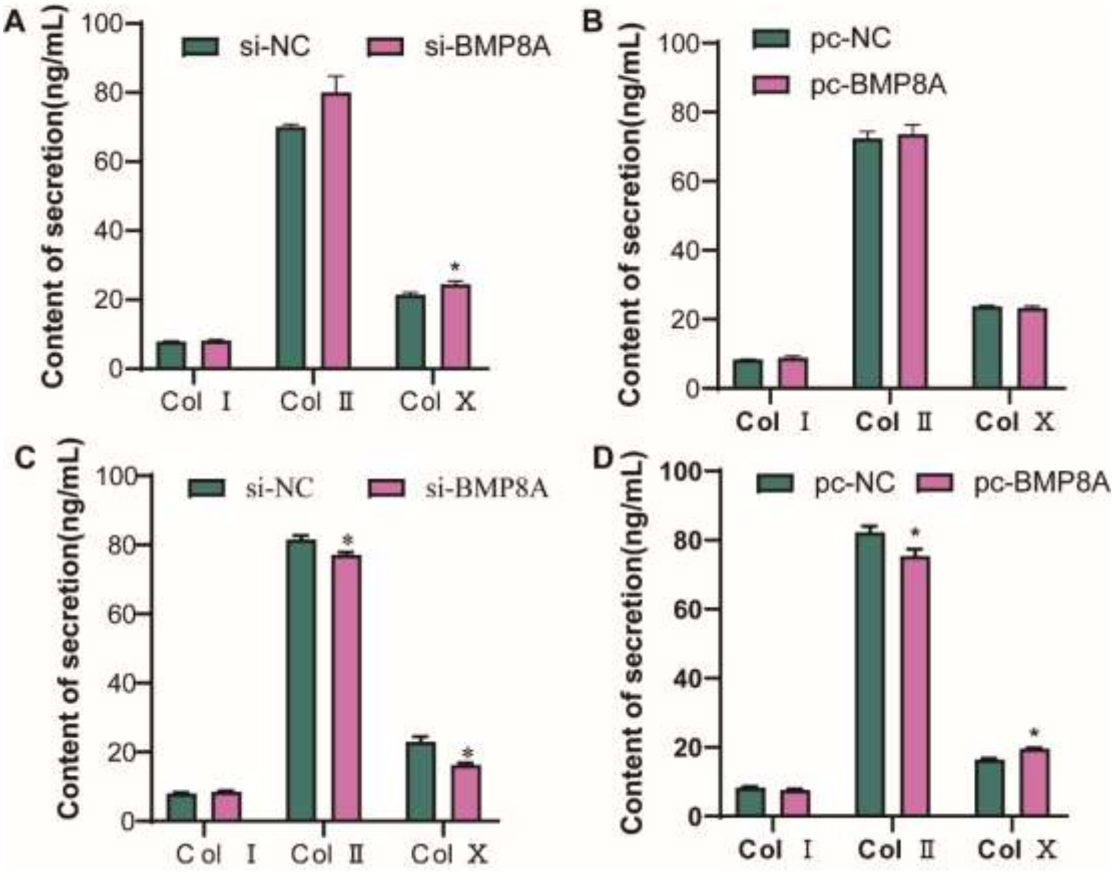

Supplement 14. Effect of BMP8A on special protein secretion of chicken chondrocytes. The protein expression of Col I/II/X were determined by ELISA in the control (A, B) and TD (C, D) chicken chondrocytes supernatant after transfection of BMP8A. All data was presented as mean±SEM for n=3 (Values represent the mean of three technical replicates), * $P<0.05$.
